# Label-free spatiotemporal decoding of single-cell fate via acoustic driven 3D tomography

**DOI:** 10.1016/j.mtbio.2024.101201

**Published:** 2024-08-13

**Authors:** Yuxin Wang, Shizheng Zhou, Yue Quan, Yu Liu, Bingpu Zhou, Xiuping Chen, Zhichao Ma, Yinning Zhou

**Affiliations:** a Joint Key Laboratory of the Ministry of Education, Institute of Applied Physics and Materials Engineering, University of Macau, Avenida da Universidade, Taipa, Macau, 999078, China; b State Key Laboratory of Quality Research in Chinese Medicine, Institute of Chinese Medical Sciences, University of Macau, Avenida da Universidade, Taipa, Macau, 999078, China; c Institute of Medical Robotics, School of Biomedical Engineering, Shanghai Jiao Tong University, No.800 Dongchuan Road, Shanghai, 200240, China

**Keywords:** Acoustic-induced vibration, Single cell rotation, Deep learning, 3D tomography, Cell fate projection

## Abstract

Label-free three-dimensional imaging plays a crucial role in unraveling the complexities of cellular functions and interactions in biomedical research. Conventional single-cell optical tomography techniques offer affordability and the convenience of bypassing laborious cell labelling protocols. However, these methods are encumbered by restricted illumination scanning ranges on abaxial plane, resulting in the loss of intricate cellular imaging details. The ability to fully control cellular rotation across all angles has emerged as an optimal solution for capturing comprehensive structural details of cells. Here, we introduce a label-free, cost-effective, and readily fabricated contactless acoustic-induced vibration system, specifically designed to enable multi-degree-of-freedom rotation of cells, ultimately attaining stable in-situ rotation. Furthermore, by integrating this system with advanced deep learning technologies, we perform 3D reconstruction and morphological analysis on diverse cell types, thus validating groups of high-precision cell identification. Notably, long-term observation of cells reveals distinct features associated with drug-induced apoptosis in both cancerous and normal cells populations. This methodology, based on deep learning-enabled cell 3D reconstruction, charts a novel trajectory for groups of real-time cellular visualization, offering promising advancements in the realms of drug screening and post-single-cell analysis, thereby addressing potential clinical requisites.

## Graphical abstract

**Figure:**
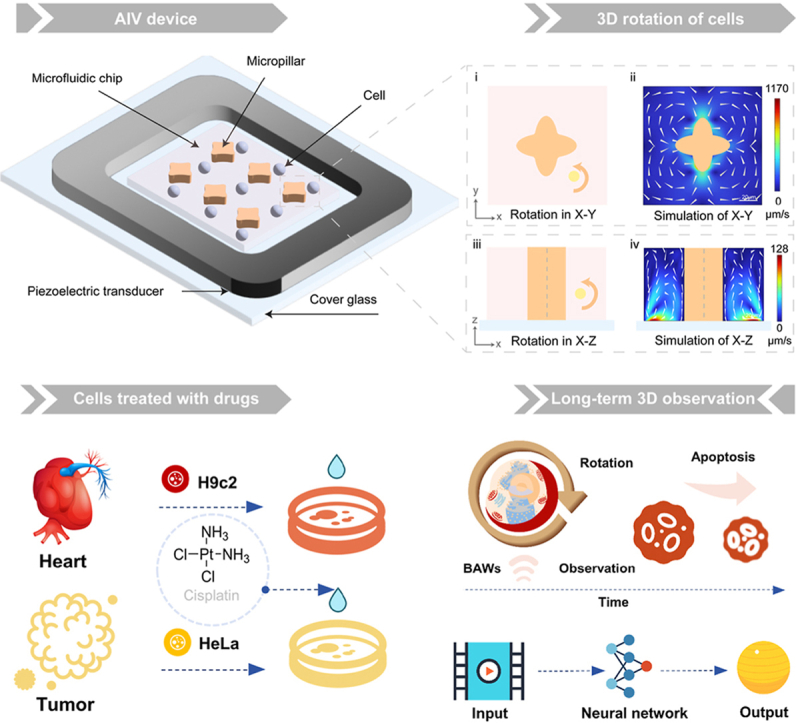

## 1 Introduction

Single-cell analysis contributes to elucidating the intricacies of cellular interactions [[1], [2], [3]], uncovering disease pathways [4,5], and assessing therapeutic responses [6,7], providing essential insights into biomedical research from a microscopic viewpoint [8]. Compared to various techniques such as ultrasound imaging [9], X-ray imaging [10], MRI [11], electron microscope [12], atomic force microscopy [13], optical imaging stands out as the most accessible for real-time cellular observation [14], ideal for dynamic cellular physiology visualization.

State-of-the-art optical microscopy techniques [[15], [16], [17]] have ascended as potent tools for the detailed study of three-dimensional (3D) structural and functional properties of living cells with high specificity. More recently, the emergence of 3D optical tomography [18] has gained prominence as a label-free microscopic technique [19,20], offering high-resolution and long-term observation [21] of single-cell dynamics without dependency on exogenous labelling agents. Typically, 3D optical tomography [22,23] employs illumination tomography to acquire two-dimensional projections at various orientations [24]. These projections are then processed using sophisticated algorithms [25] such as filtered back projection (FBP) [26] and algebraic reconstruction technique (ART) [27] to achieve 3D reconstruction. Nevertheless, the limited numerical aperture [28] of microscope objectives presents a challenge, constraining the range of light angles captured during tomography. This limitation results in a lower resolution in the vertical direction compared to the lateral direction, a phenomenon known as the missing cone problem [29]. To overcome this, full-angle tomography of cells in 3D space becomes imperative [30]. This approach aims to gather comprehensive information about cellular structures, providing prior knowledge for subsequent algorithms, and thus ensuring more accurate and complete 3D reconstruction.

Recent advancements in cell rotation strategies, particularly those based on microfluidics with advantages including high-precision manipulation and real-time monitoring [31], have shown highly promise in the capability for full-angle tomography of cells. A variety of techniques for manipulating cell rotation have been developed, ranging from mechanical contact methods [32] to non-contact approaches supported by optical [33,34], electrical [[35], [36], [37]], magnetic [38], acoustic fields [[39], [40], [41], [42], [43]], etc. Among these, the utilization of acoustic-induced vibration (AIV) [44,45] stands out for its excellent biocompatibility and significant reduction in equipment costs. AIV, which employs acoustic phenomenon such as acoustic radiation force, acoustic cavitation, and acoustic streaming [46], allows for precise control of cell rotation in 3D space [47]. While most AIV-based strategies have been concentrated on local in-plane rotations employing X or Y-axis vibrations, there is less emphasis on combining these with precisely controllable out-of-plane rotation, which is induced by Z-axis vibration, to visualize the complete structural information [48]. This exploration of multi-degree-of-freedom rotation using AIV for full-angle tomography is a crucial step towards achieving precise 3D cell reconstruction [49]. However, it is worth noting that as the rotational degrees of freedom increase, so too does the size of the datasets acquired from 3D optical tomography. The challenge then lies in the process of these large datasets [50] for 3D reconstruction, which involves complex computational steps and high computational costs. Over the past few decades, artificial intelligence (AI) [51], particularly deep learning (DL) [8,52,53], and computer vision technologies [54,55] have undergone revolutionary advancements [56], significantly propelling the field of 3D reconstruction forward. This progress has facilitated the processing of large image datasets obtained from microscopy, enabling achievements such as super-resolution reconstruction [57], precise cell segmentation [58], enhanced pseudo color rendering [59], accurate pattern recognition [60], etc.

Innovations in AI technology have enhanced the foundational work in cellular biology techniques. Based on CNN, Kusumoto et al. [61] developed the Deep-SeSMo and Pattarone et al. [62] trained models for classification of brightfield images. With label-free advantage, Christiansen et al. [63] introduced ISL and Hartnett et al. [64] developed the LANCE. Focused on morphological features, Blasi et al. [65] showcased machine learning based on images of cells using imaging flow cytometry. Nevertheless, the aforementioned studies predominantly utilize 2D cellular images. Investigations that employ full-angle video recordings via 3D cell rotations as input data for deep learning, aimed at extensively observing the spatiotemporal fate of cells, are yet to be conducted.

In this study, we present a long-term and label-free platform for spatiotemporal observation of cell fate, leveraging DL in conjunction with multi-degree-of-freedom rotation of single cells facilitated by an ultralow-cost hyperstatic AIV system. This novel approach effectively bridges the gap between holographic AIV-based uniform single-cells rotation and DL-driven 3D reconstruction techniques. Our method employ a microfluidic chip, innovatively designed with arrayed four-pointed stars, to achieve acoustic control of full-angle cell rotation. This design allows for stable rotation both in-plane and out-of-plane, essential for comprehensive 3D imaging. Four diverse cell types were adopted to validate the system performance stability, utilizing a series of computer vision algorithms for detailed morphological analysis. After the feature extraction, we employ principal component analysis (PCA) and two DL algorithms to process small samples and large samples respectively for the accurate identification of cell types. Remarkably, our optimized algorithms demonstrate extremely high accuracy in tasks of classification. Additionally, a DL framework is designed based on 3D convolutional neural networks (3D-CNN), specifically utilized for single-cell 3D reconstruction. This original approach not only achieves precise structural reconstruction of cell nuclei and membranes across four various cell types but also facilitates long-term, minute-frequency observation of groups of single cellular changes. Overall, our DL-driven workflow, built upon full-angle rotation scanning within a uniform and hyperstatic microfluidic environment, not only enhances our capacity to observe and comprehend the fate of single cells but also promises to establish a new paradigm for biomedical research focused on single-cell analysis.

## 2 Materials and methods

### 2.1 Chip fabrication

The chip mold for the arrayed four-pointed stars chip was initially fabricated using standard soft lithography procedures, employing a silicon/SU8 master. Subsequently, a mixture of Sylgard 184 silicone elastomer base and curing agent (Dow Corning, Midland, MI, USA) was prepared at a ratio of 10:1 (w/w) for PDMS (Fig. S1c) chip fabrication. As shown in Fig. S1d, the prepared mixture was then cast onto the photolithographic chip mold. Following degassing under vacuum, the entire device underwent baking at 80 °C for 2h. The cured PDMS array was delicately detached from the chip mold. Following this, both the PDMS array and a cover glass were subjected to a 3-min treatment with oxygen plasma (PDC-002, Harrick Plasma, USA), generating hydroxyl functional groups on the surfaces. The treated surfaces contacted with each other, forming enclosed microfluidic channels.

In this experiment, the four-pointed star pattern could be regarded as being composed of two identical orthogonally intersecting ellipses, with their centers overlapping. Each ellipse had a major axis length of 60 μm μm and a minor axis length of 20 μm. In the array, the centers of adjacent ellipses, both horizontally and vertically, were spaced 180 μm apart. The pillar height was 60 μm.

### 2.2 Experimental setup

The above microchip was secured on a piezoelectric transducer (P16.XY20Z7K, COREMORROW, China). A function generator (AFG 31102, Tektronix, USA) introduced sinusoidal input signals along the X/Y or Z-axis, which were amplified through a piezoelectric controller (E00.A4, COREMORROW, China), and then input into the piezoelectric transducer, generating vibrations along the X/Y or Z direction. The assembled device was mounted on an inverted optical microscope system (IX73, Olympus Corporation, Japan), which was equipped with a digital camera (DP74, Olympus Corporation, Japan) for observation of the conduction of the experiments. The schematic diagram of this system is shown in Fig. 2a. In rotational recording, optimizing the rotation speed was essential for balancing high-quality reconstruction with practical constraints, such as extended imaging times and system stability. We employed a method that adjusted the speed by modulating the frequency of acoustic vibrations, using simulations and experimental validations in the 500–2500 Hz range. We confirmed that 1500 Hz could stably induce rotation while precisely preserving surface detail information. Our approach was designed to efficiently navigate these trade-offs, ensuring that we delivered accurate and reliable reconstructions.

For generating output labels for 3D reconstruction with the 3D-CNN regression algorithm, authentic 3D cellular data were preemptively captured using confocal microscopy (LSM710, Carl Zeiss, Germany). After training the neural network model, video data of cell rotation, captured using a standard microscope's bright field, are reintroduced to the model. This process enables direct regression of 3D cell reconstruction data, bypassing the need for confocal microscopy (Fig. 3, Fig. 5c).

### 2.3 Cell culture and preparation

The experiment utilized cells including HeLa (Henrietta Lacks cells), B16 (B16 melanoma cells), H9c2 (cardiac myoblasts), and MP (macrophages). These cells were cultured in Dulbecco's Modified Eagle's Medium (DMEM, Gibco, USA) supplemented with 10 % fetal bovine serum (FBS, Gibco, USA) and 1 % penicillin/streptomycin (15140-122, Gibco, USA). Cells were maintained at 37 °C, 5 % (v/v) CO_2_ in a cell incubator (BPN-40RH, Bluepard, China). To obtain a cell suspension, adherent cells were washed twice with 2 ml of PBS and then treated with 0.5 ml of 0.25 % trypsin-EDTA (25200-056, Gibco, USA) for 1 min. Subsequently, 2 ml of the medium was added to deactivate the trypsin. Finally, the solution was centrifuged using a centrifuge (TDZ5-WS, CenLee, China) at 350 g for 5 min to increase the cell concentration. The supernatant containing trypsin was discarded, and 1 mL of PBS was added to the tube. The cells were diluted to concentrations around 2 × 10^5^ cells/ml.

For the rotation experiments, adherent cells were freshly taken out from the incubator and promptly introduced into a microfluidic device, where they were exposed to acoustic-induced vibrations. Cellular rotation was monitored using a microscopy, and video data were captured, encompassing 310 frames across a 15-s interval. It is imperative to highlight that the entire process, from the cell digestion to suspension and the completion of filming, was rigorously completed within 5 min. This rapid execution of the experimental protocol substantially alleviates concerns related to cell viability during extended periods of suspension.

### 2.4 3D-CNN for reconstruction

In this paper, we constructed a 3D convolutional neural network. For the training of the 3D Convolutional Neural Network (3D-CNN), we utilized video data that captured the acoustic-induced rotation of cells. Each video frame was processed to convert it into a two-dimensional matrix. We enhanced the focus on individual cells by selecting the largest bounding rectangle for each cell as the Region of Interest (ROI). These matrices were subsequently stacked over time, creating a three-dimensional matrix, with each element stored as a double data type. The output data for the 3D-CNN training were sourced from confocal microscopy images. In a manner akin to the input processing, we extracted two-dimensional slice data of cells, isolated the largest bounding rectangle as the ROI for each cell, and compiled these slices along the Z-axis to construct another three-dimensional matrix, which was also maintained in double format. This methodology ensured precise spatial alignment and data integrity essential for effective 3D-CNN training.

The 3D-CNN regression algorithm used convolutional layers to extract spatial features, the Rectified Linear Unit (ReLU) [66] activation for non-linearity, and a regression layer to map the features to the final output. The network was trained to minimize the difference between predicted and actual regression values using a suitable loss function.

Firstly, we created an input layer with dimensions of 100 × 100× 10 pixels. Then, the 3D convolutional layer captured spatial hierarchies and patterns in the input volume using convolutional filters. The filters slid over the input, and the dot product of filter weights and input values was computed at each position. The output of a 3D convolutional layer could be calculated using the following formula:

(1) $$Z_{ijk}=\sum_{l=1}^{L}\sum_{m=1}^{M}\sum_{n=1}^{N}W_{lmn}\cdot X_{\left(i+l\right)\left(j+m\right)\left(k+n\right)}+b$$

Where: $Z_{ijk}$ is the output at position (i,j,k), $W_{lmn}$ are the weights of the filter, $X_{\left(i+l\right)\left(j+m\right)\left(k+n\right)}$ is the input at position (i+l,j+m,k+n), *b* is the bias term. In this study, we constructed a five-layer convolutional network, each layer with a filter size of 3 and filter numbers of 64, 32, 16, 8, and 4, respectively. Each layer had dimensions of 3 × 3× 3 with a stride of 1 applied in all directions. The weights were defined by five 3D matrices, each with a first and second dimension of 3. The third dimension of these matrices aligned with the filter numbers across the five layers. The biases were encapsulated in five 3D matrices, where the first two dimensions of each matrix were 1, and the third dimension matched the number of filters present in each of the five layers.

Subsequently, the ReLU activation introduced non-linearity by setting all negative values to zero. This helped the network learn complex relationships in the data. The ReLU activation function was applied elementwise to the output of the convolutional layer:

(2) $$A_{ijk}=\max\left(0,Z_{ijk}\right)$$

Where: $A_{ijk}$ is the output of the ReLU activation.

Finally, the regression layer combined the information learned by the previous layers to produce the final regression output. It applied a linear transformation to the features obtained from the convolutional layers and ReLU activation.

## 3 Results and discussion

### 3.1 Single cells behaviour capturing through AIV system with ultralow frequency

**Fig. 1:**
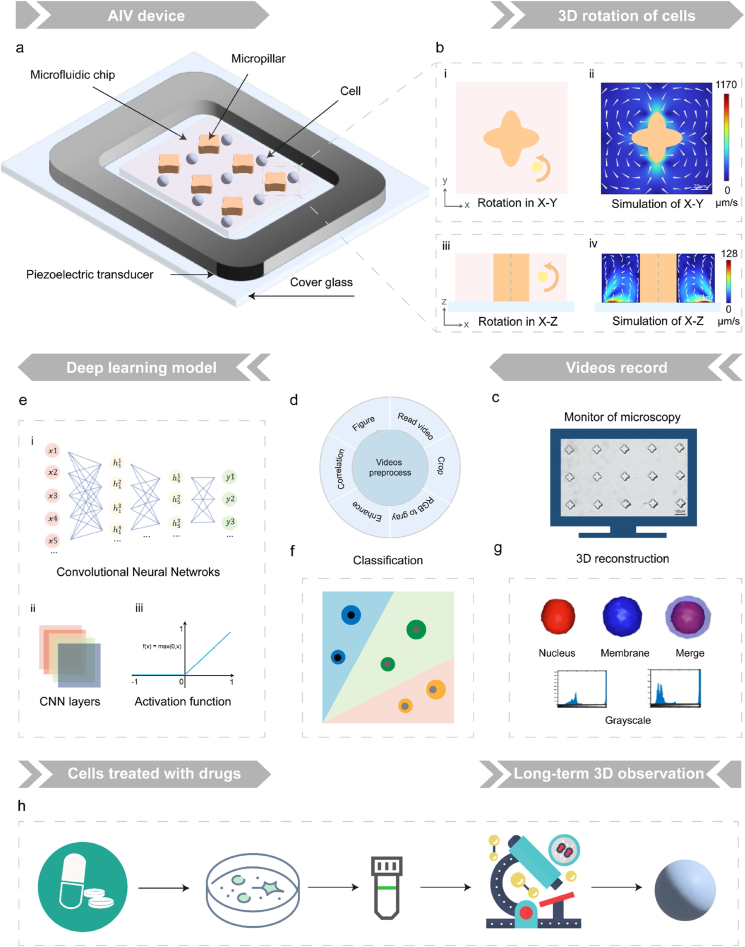
Principle of the AI-driven long-term 3D cell tomography system. **a)** Conceptual sketch of the acoustic-induced vibration (AIV) platform. It composes a piezoelectric transducer and a microfluidic chip coupled onto a cover glass. **b)** Schematic diagrams of two rotation modes and their numerical simulations. i-ii) In-plane rotation occurs when there's vibration along the X-axis. iii-iv) Out-of-plane rotation happens with Z-axis vibration. Scale bar: 20 μm **c)** Observation of cell rotation using an inverted microscope. **d)** Video pre-processing workflow. **e)** Deep learning framework of 3D reconstruction. i) An overview of the convolution neural network (CNN) architecture. ii) Explanation of the CNN layers. iii) The ReLU activation function. **f)** Diagram of classification among various cell types. **g)** Illustration of the 3D reconstruction of cell nucleus and membrane, accompanied by grayscale distribution histograms. **h)** Diagram of long-term observation of drug-treated cells.

Achieving stable in-situ cell rotation is essential for the accuracy of 3D tomography. In response, an ultralow-cost, hyperstatic AIV system has been implemented. Fig. 1a shows the system design principle: the piezoelectric transducer, innovatively manufactured with high-reliability ceramics and flexible hinges, enables high-resolution, rapid-response vibrations along the X/Y/Z-axis. Upon initiating linear vibration, the piezoelectric transducer causes the attached microchip to vibrate in unison. The harmonized vibration is efficiently conveyed to the surrounding fluid via the micropillar arrays of the microchip. Consequently, vibration energy dissipates into the fluid's boundary layer near the micropillars, creating steep velocity gradients and intense flow, known as inner boundary layer streaming [67]. Based on our previous research [[44], [45], [46]], a four-pointed star array has been developed (Figs. S1a–b) to facilitate precise 3D rotation of individual cells with AIV streaming generated within respective planes, as elaborated in Section 2.1 and Fig. 1. Moreover, the parameters for optimized stable in-situ rotation control of single cells were further investigated using simulation and experimental techniques.

**Fig. 2:**
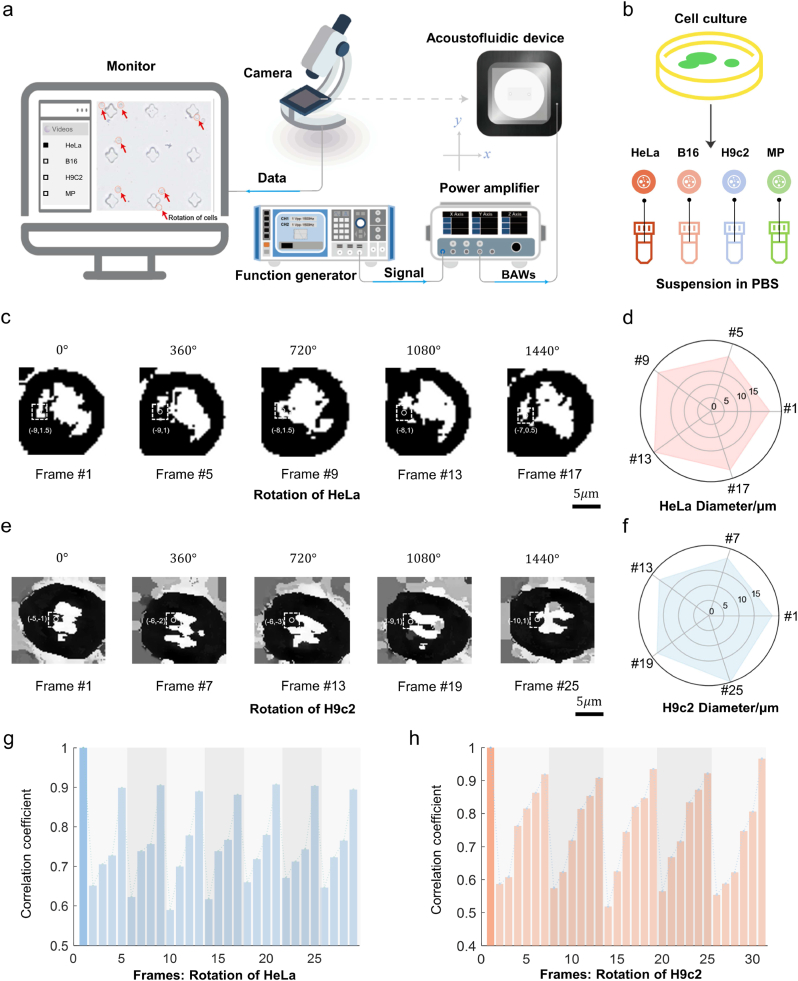
Characterization of in situ and stable rotation of cells controlled by AIV. **a)** Monitor of the rotation position and the platform configuration. A signal generator emits an electrical signal. Then a piezoelectric transducer converts it into bulk acoustic waves (BAWs) that induce cell rotation within the microfluidic chip. **b)** Biological sample preparation: HeLa (Henrietta Lacks cells), B16 (B16 melanoma cells), H9c2 (cardiac myoblasts), and MP (macrophages). **c)** Out-of-plane rotation of HeLa cells induced by Z-axis vibration is recorded, with feature points tracked in videos. Images are captured at each 360° rotation increment. Scale bar: 5 μm. **d)** Major axis diameter analysis of five frames from c) is presented in a radar chart, with frame numbers as axis labels and cell diameters as data points. **e)** Out-of-plane rotation of H9c2 cells induced by Z-axis vibration is recorded, with feature points tracked in videos. Images are captured at each 360° rotation increment. Scale bar: 5 μm. **f)** Major axis diameter analysis of five frames from e) is presented in a radar chart, with frame numbers as axis labels and cell diameters as data points. **g-h)** Frame-to-frame correlation analysis chart. For both HeLa and H9c2 cells during rotation, the subsequent images are correlated with the first image to determine their correlation coefficients. Two bar charts showing the periodic changing of the correlation coefficients over time are plotted separately for each cell type.

**Fig. 3:**
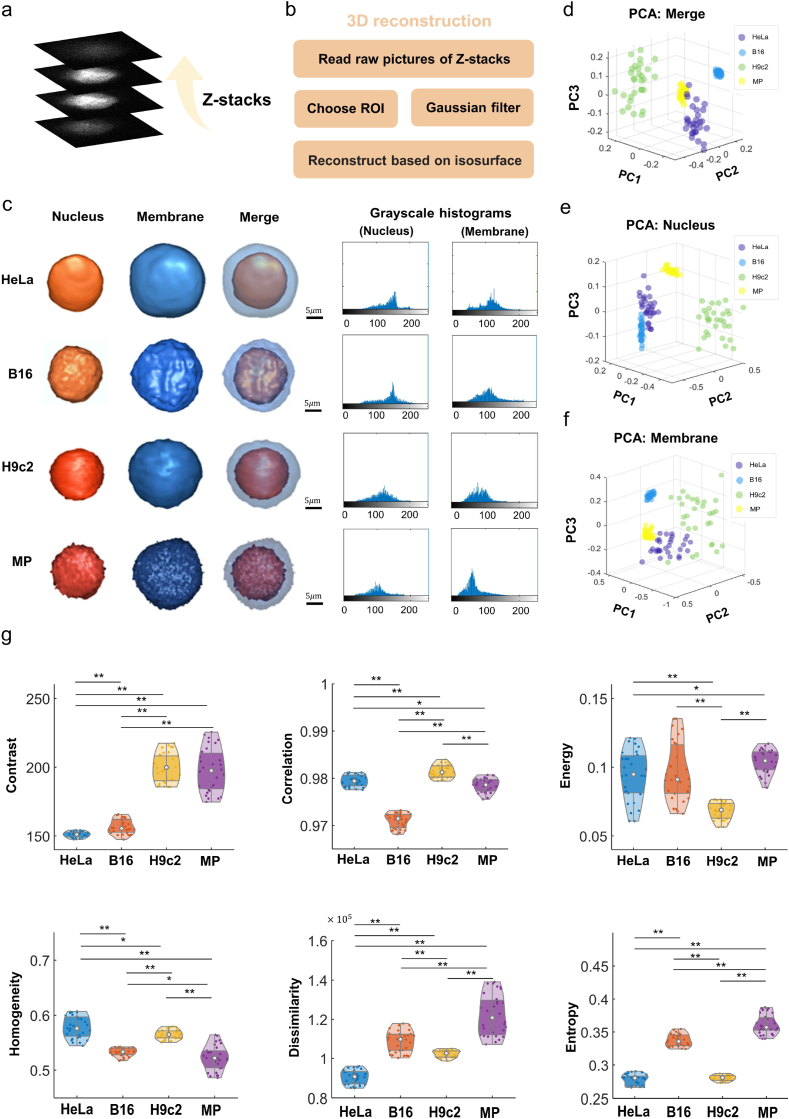
3D reconstruction and data analysis for heterogeneous cells. **a)** Schematic of optical tomography: utilizing collected stacks of confocal two-dimensional slice data to perform 3D reconstructions **b)** Descriptions of the 3D reconstruction methodologies employed. **c)** 3D reconstruction outcomes (Scale bar: 5 μm) and gray-scale histograms. **d-f)** 30 times 3D reconstruction for statistical robustness, with principal component analysis (PCA) utilized to ascertain the first three components highlighting key features. **g)** Quantitative analysis through violin plots. These plots depict attributes of gray-level co-occurrence matrix (contrast, correlation, energy, homogeneity, dissimilarity, entropy) across 30 iterations of 3D reconstruction. Each plot represents the four cell types, annotating significance with ** for p-value <0.001 and * for 0.001 < p-value <0.05, as determined by Student's t-test. N number = 30.

To prevent trajectory capture issues due to excessive cell movement speeds from high voltage amplitudes, an extremely low peak-to-peak voltage of 1Vpp was utilized. Simulations of X-axis and Z-axis vibrations with frequency screening from 500 Hz to 2500 Hz were conducted (Fig. S2). The color bar shows the flow velocities' values, and white arrows point out the flow directions. The simulation results indicate that by adjusting the vibration frequency, the rotation speed can be controlled. In simulation of vibration along the X-axis, vortices are virtually undetectable at both 500 and 1000 Hz. At 1500 Hz, however, vortices with high circularity and uniform velocities along their circumferences become evident. At higher vibration frequencies of 2000 Hz and 2500 Hz, markedly vigorous vortices are observed. These currents exhibit significant acceleration at the column's sharp corners, indicating cell instability in their velocity in a complete rotation. In parallel simulation of Z-axis vibration, the formation of vortices at 500 and 1000 Hz is negligible, with 1500 Hz showing vortices of greater circularity compared to those at 2000 Hz and 2500 Hz. Notably, at 2000 Hz and 2500 Hz, the vortices deviate from axial rotation, instead of an in-situ rotation. Moreover, given considerations of lower power consumption, 1500 Hz is identified as the optimal frequency from these conditions. Fig. 1b and S2 simulation results demonstrate that at a 1Vpp amplitude and 1500Hz frequency, applying vibration in the X-axis generates four symmetric, circular vortices around a single four-pointed star pillar. The centers of these circular vortices align with the four-pointed star's center at 45° angles to the X or Y-axis, at a Euler distance about 30 μm. In the simulation of Z-axis vibration, the observation plane is created by rotating the X-Z plane 45° around the Z axis. Two vortices appear on the pillar's lower left and right sides, each centered about 50 μm perpendicular from the pillar's central axis and about 10 μm above its base.

Fig. 1 offers a thorough overview of the working philosophy described in this paper. In addition to the core of the AIV system (Fig. 1a) and the representative simulated cell rotation modes (Fig. 1b) mentioned above, the concept diagram also illustrates procedures including: cell rotation videos obtaining under a microscope (Fig. 1c), processing data using computer vision algorithms (Fig. 1d) and reconstructing the 3D cell structure using neural network models automatedly after video data acquiring (Fig. 1e–g). Furthermore, various AI techniques are utilized to classify and analyze diversified 3D cell data (Fig. 1f). Videos depicting various drug-induced cell apoptosis are also collected and incorporated into a neural network model to analyze the precise spatiotemporal dynamics of cellular fate. (Fig. 1h). Next, we will provide detailed elaboration on the results and discussion of the experiments.

### 3.2 Various single cells dynamic in situ 3D precise controlling

Firstly, the results related to precise controllable 3D cell rotation are presented. After setting up the AIV system, cells were injected into the microchip. Upon activating the signal generator, we observed stable and in-situ rotation of cells at the inner corners of the micropillars. Connecting the piezoelectric transducer to the X/Y-axis vibration channel induces in-plane rotation of cells, while connecting it to the Z-axis vibration channel causes out-of-plane rotation of cells, consistent with simulation outcomes. Notably, nearly all individual patterns capture at least one cell (Fig. 2a), enabling uniform and simultaneous rotation of multiple single cells in large scales, thereby significantly enhancing experimental efficiency. This approach offers new perspectives on the precise and uniform manipulation of cell rotation on a large scale, facilitating high-throughput operations.

To validate the applicability of our AIV system across broad cell types, we conducted experiments involving four cell types (Fig. 2b). Subsequently, cell rotation videos were captured at a frequency of 1500 Hz, and various analytical methods were employed to assess the stability of cell rotation. These methods included template matching, diameter measurement, and calculation of correlation coefficients to monitor periodic changes. For off-axis evaluation, a specific feature point was identified in the initial frame and tracked across 1, 2, 3, and 4 rotations (Fig. 2c and e, S3). For instance, in the case of out-of-plane rotation of HeLa (Henrietta Lacks cells), the angle deviations for the identified feature points were computed as 0.16°, 0.11°, 0.18°, and 0.07°, indicating a high level of rotational consistency. Furthermore, we employed a sequence of algorithms, including image binarization, morphological dilation, and automatic circle detection, to quantify changes in the major axis lengths of cells across five images. Radar charts were then generated to visualize these variations for the four cell types (Fig. 2d and f, S4). Subsequently, correlation coefficients between the initial frame and subsequent frames were calculated, and histograms of these coefficients were plotted to detect periodic changes (see Fig. 2g and h, S5). Collectively, these findings provide evidence supporting the precise control of cell rotation achieved by our AIV system.

### 3.3 Single cells 3D tomography and precise classification

Following the acquisition of stable multi-degree-of-freedom rotation videos of cells, output labels for subsequent supervised DL-based 3D reconstruction were finalized. Initially, 3D tomography was conducted on each of the four cell types using confocal microscopy, as depicted in Fig. 3a. Subsequently, acquired data were input into MATLAB software for 3D reconstruction, following the method outlined in Fig. 3b. 3D images of the cell nuclei and cell membranes for the four cell types were then reconstructed (Fig. 3c–S6-9). Observable diversities among the four cell types are evident in these pictures. Next, we delve into the physiological mechanisms behind these features in detail.

Firstly, striations can be observed on the surface of H9c2 (cardiac myoblasts), which may be due to muscular characteristics. The unique muscular traits of H9c2 stem from the production of essential proteins critical for contraction and relaxation, notably calcium pump proteins like SERCA [[68], [69], [70]]. Moreover, we find the macrophages (in this article, abbreviated as MP) have rougher surface and numerous protrusions, which could be key to their roles in the immune system, aiding in phagocytosis and the clearance of pathogens and debris. These protrusions, known as pseudopodia, allow MP to envelop and ingest targets. Their formation is regulated by proteins such as integrins and CD44, highlighting their critical function in immune responses [71,72]. Furthermore, HeLa exhibit smooth characteristics, which may result from to their epithelial nature and cytoskeletal structure. The dense arrangement of epithelial cells and their tight connections result in a flat cell surface. The cytoskeleton, consisting of microfilaments, intermediate fibers, and microtubules, underpins the cell's structure and contributes to the smooth cell surface morphology [68,73]. Meanwhile, observations indicate that the surface texture of B16 (B16 melanoma cells) is notably rougher when compared to HeLa. As a melanoma cell line, B16 cells are characterized by a greater number of protrusions and a more irregular shape than epithelial cells. This distinction could stem from their heightened motility and invasive characteristics, likely linked to the expression of collagenase and integrins in B16 [74].

To conduct a quantitative analysis of the differences in 3D structures, grayscale co-occurrence matrices were generated from the 3D images of four distinct cell types. These matrices facilitated the derivation of six morphological parameters: contrast, correlation, energy, homogeneity, dissimilarity, and entropy. Following this, the PCA [75] algorithm was employed to identify the three most significant principal components. This step enabled the creation of 3D scatter plots (illustrated in Fig. 3d–f), which effectively highlight the distinguishability among the four cell types. Additionally, violin plots (shown in Fig. 3g) were produced to offer a quantitative insight into the morphological characteristics of the cells.

To further evaluate the feature differences among the four types, a machine learning method, support vector machine (SVM) [76], suitable for small sample data was employed. The SVM model used sequences of one-dimensional grayscale distributions for four-class classification. The leave-one-out method was applied to divide the datasets into training and test sets. Then, we computed the confusion matrix and calculated precision, recall, F1-score, and accuracy, obtaining a 90 % accuracy (Fig. S12). In order to avoid the high error rate caused by the small datasets, traditional data augmentation and denoising diffusion probability model (DDPM) [77] were utilized for data augmentation (Fig. S10), which reduced the impact of misclassified images on the overall accuracy. It is worth noting that DDPM is an algorithm that learns the original feature distribution to generate a brand-new image, which can avoid the model overfitting caused by traditional data augmentation to a certain extent. We applied data augmentation to the original training set and test set respectively, and finally obtained 800 images for training and 200 for testing. The DL model was trained based on ResNet50 [78] with a 5-fold training approach and evaluated on an independent test set. The model's classification accuracy exceeds 98 % in the classification task for four cell types (Figs. S11–12). The experiments confirm that training DL models with data augmentation methods substantially improves accuracy. These methods stand apart from conventional strategies for cell characterization and classification that primarily utilize two-dimensional images. By leveraging a 3D perspective on cell images and applying AI technologies, it substantially augments the 3D cell image database. This approach introduces a novel paradigm for achieving precise cell classification.

### 3.4 3D–CNN–regression reconstruction of cells

After obtaining the output labels for reconstruction, we proposed an end-to-end supervised learning method of regression, 3D-CNN [[79], [80], [81]], for reconstruction of cells. This method takes rotation videos under bright-field microscopy as the inputs and the outputs are stacks of two-dimensional slices. Upon successful training of the neural network model, it becomes capable of directly reconstructing the 3D structure of cells solely from bright-field videos, significantly enhancing applicability across diverse scenarios, raw laboratory environments, and a wide range of targets.

**Fig. 4:**
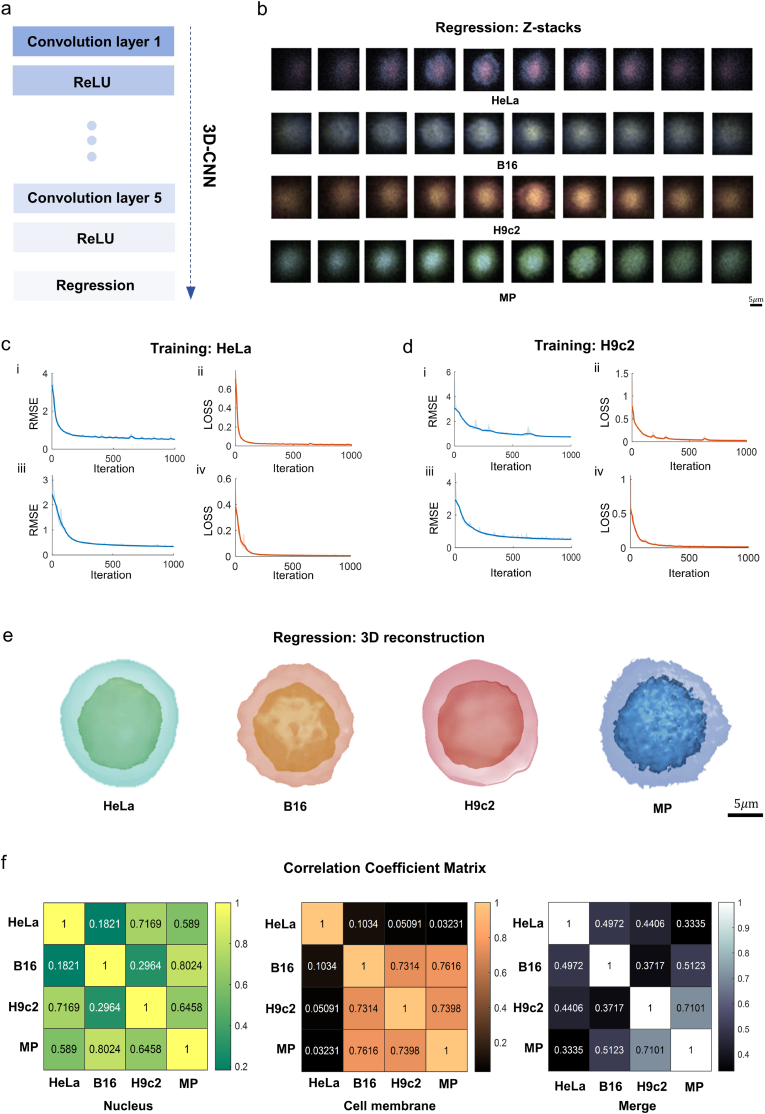
3D-CNN based regression and analysis. **a)** The CNN architecture depicts a 3D-CNN consisting of seven convolutional layers, each incorporating a rectified linear unit (ReLU) activation function, ultimately leading to a regression output layer. **b)** Z-stack slices processed through the 3D-CNN of four cell types (HeLa, B16, H9c2, MP) and the use of pseudo-colors for each cell type, prepared for subsequent 3D reconstruction. Scale bar: 5 μm. **c)** HeLa cells training progress. The picture illustrates the training progression of root mean squared Error (RMSE) and loss, subdivided into cell nucleus analysis i-ii) and cell membrane analysis iii-iv). **d)** H9c2 cell training progress. It displays the RMSE and loss development over training session, subdivided into cell nucleus analysis i-ii) and cell membrane analysis iii-iv). **e)** Predictive 3D reconstruction outcomes. They exhibit the 3D reconstruction predictions for the four cell types by the 3D-CNN framework. Scale bar: 5 μm. **f)** Inter-cell type cross-correlation analysis. It presents matrices of cross-correlation coefficients among the four cell types, subdivided into nucleus, membrane, and combined nucleus and membrane analyses. (For interpretation of the references to color in this figure legend, the reader is referred to the Web version of this article.)

Firstly, the videos were parsed into a sequence of images (including videos of rotation in-plane and out-of-plane rotation). 30 samples were selected for each of the four cell types, and a separate model was trained for each cell type, yielding a total of four models. Each model underwent 1000 training epochs with a five-layer neural network (Fig. 4a). As shown in Fig. 4c–d, and S13-14, the RMSE and loss metrics for all four models exhibit a decreasing and converging trend, indicating the good and effective model performance on the training set. Subsequently, the network performance was tested on the test set by inputting another batch of rotation video data. Each of these models output a set of two-dimensional slice stacks (Fig. 4b), which were then subjected to 3D reconstruction using the isosurface method. The reconstruction is shown in Fig. 4e. The 3D reconstruction results of the regression display the same morphological features as the 3D reconstruction used as model labels in Section 3.3. In order to compare inter-class differences, correlation coefficients are computed for the 3D reconstruction images of the cell nuclei, cell membranes, and their combination for the four cell types, resulting in three correlation coefficient matrices (Fig. 4f). The matrices reveal weak similarities for cell membranes and cell nuclei, and even weaker similarities for the combined 3D reconstruction of cell nuclei and membranes (83.3 % of the correlation coefficients below 0.5). This indicates that our model trained above demonstrates robustness and exhibits distinct inter-class differences. It provides a new approach for cell 3D reconstruction, enabling low-cost, label-free, and high-precision observation of dynamic 3D changes in cells, laying the foundation for applications such as long-term observation of living cells.

**Fig. 5:**
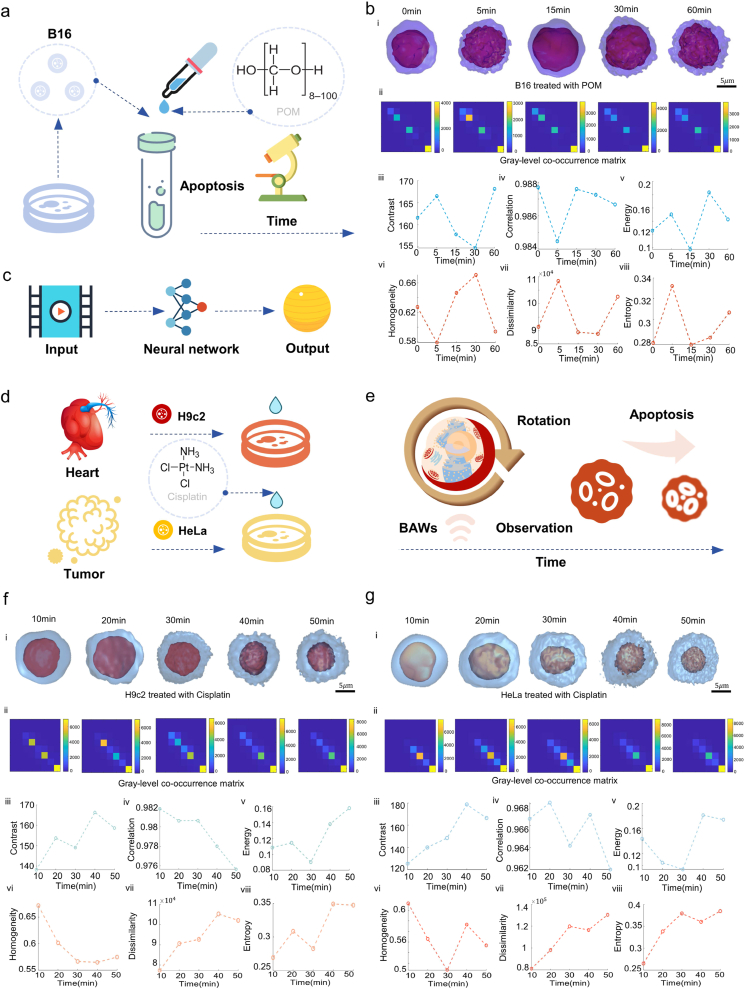
Monitoring spatiotemporal alterations in cellular architecture with drug exposure. **a)** Diagram of B16 cell apoptosis. **b)** Apoptosis monitoring. i) Sequential visualization of B16 cells treated with POM, showcasing 3D structural transformations at 0, 5, 15, 30, and 60 min. Scale bar: 5 μm. ii) Gray-level co-occurrence matrix analysis for cellular structures at these time intervals. iii-viii) Temporal evolution line charts for contrast, correlation, energy, homogeneity, dissimilarity, and entropy derived from the gray-level matrices. **c)** Diagram of the end-to-end neural network regression for 3D reconstruction. **d)** Cisplatin treatment: H9c2 and HeLa cells. **e)** Schematic diagrams of the drug-exposed cells during rotation at various time to observe the process of cell apoptosis. **f)** Cisplatin-induced apoptosis in H9c2 Cells. i) Depicting 3D structural shifts at 10, 20, 30, 40, and 50 min. Scale bar: 5 μm. ii) Gray-level co-occurrence matrix analysis at these time points. iii-viii) Line graphs in contrast, correlation, energy, homogeneity, dissimilarity, and entropy from the matrices. **g)** Cisplatin-induced apoptosis in HeLa cells. i) Depicting 3D structural changes at 10, 20, 30, 40, and 50 min. Scale bar: 5 μm. ii) Gray-level co-occurrence matrix evaluation at these intervals. iii-viii) Line graphs for contrast, correlation, energy, homogeneity, dissimilarity, and entropy.

### 3.5 Long-term drug effect observation for single cells fate

The integration of the AIV system with the 3D-CNN method mentioned earlier has showcased significant potential for observing the long-term fate of individual cells. Diverse cell types from both cancerous and normal cell populations were further examined under various drug-induced apoptosis conditions.

Initially, we undertook an exploration of the spatiotemporal dynamics of cells, starting with one of the foundational procedures in cellular biology: cell fixation with 4 % Polyoxymethylene (POM) [82]. The objective of the fixation is to maintain the morphological integrity of cells and tissues. Nevertheless, insufficient fixation time can result in suboptimal preservation, whereas overly prolonged fixation may trigger apoptosis in cells [83,84]. Typically, an optimal fixation period is identified to be between 15 and 20 min [85]. In our research, we have innovatively explored the potential rationale for identifying the ideal fixation time, focusing on the nuanced alterations in the 3D architecture of cells as the fixation time varies (Fig. 5a). For instance, B16 cells were treated with 4 % POM. Subsequently, at 0 min, 5 min, 15 min, 30 min, and 60 min time points, real-time cell rotations were conducted using our specified AIV system. Rotation videos were then recorded at these time points and input into the model for 3D reconstruction (Fig. 5c). Furthermore, gray-level co-occurrence matrices were plotted at these five time points, and based on these matrices, we calculated the changes in six parameters (contrast, correlation, energy, homogeneity, dissimilarity, and entropy), quantitatively analyzing the fluctuating process that included information on roughness (Fig. 5b). The findings reveal that at the 0 min and 15 min intervals, the cells exhibit similar values across the six morphological parameters, a resemblance further corroborated by the visualization in 3D reconstruction images. When a live cell is initially exposed to POM, cell volume might experience minimal contraction due to osmotic pressure discrepancies between the solvent and the cytoplasmic matrix. Yet, at the widely acknowledged optimal fixation times [83,84], cellular morphology reverts to its original state. Subsequently, extending the fixation time further intensifies the cell surface's roughness, which could be attributed to the excessive protein cross-linking resulting from overly extended fixation durations. Such over-crosslinking modifies the proteins' antigenicity and 3D conformation, potentially leading to slight deformations within the cellular architecture. These modifications become evident in the altered shape of the nuclei, increased compactness of the cell membrane, and the modified structures of organelles like mitochondria and the endoplasmic reticulum [[82], [83], [84]].

Moreover, we conducted drug testing utilizing cisplatin, an anticancer drug commonly used as a first-line treatment for cervical cancer, to induce apoptosis in both cancer cells (HeLa) and healthy cells (H9c2), the 3D structural changes over time were observed to evaluate the cardiotoxicity of the drug (Fig. 5d). Following a 4-h incubation period with cisplatin drug at a concentration of 10 μg/mL (referencing the literature) [86], single cellular rotation was precisely conducted utilizing our AIV system, with rotation videos captured at 10-min intervals (see Fig. 5e). Subsequently, videos of two cell types were input into the model for 3D reconstruction (Fig. 5f and g). Gray-level co-occurrence matrices were plotted, and six morphological parameters were calculated. This provides a quantitative characterization of cell exposure to the anticancer drug from six dimensions. The ensuing examination reveals discernible 3D structural alterations concurrent with the progression of cellular apoptosis, illustrating a gradual transformation characterized by increased surface roughness and reduced cellular sizes. To investigate the specificity of cisplatin for cervical cancer cells and its cardiotoxicity towards cardiac myoblasts, the coefficient of variation (CV) of two groups of morphological parameters were calculated. CV characterizes the degree of data variability, thereby reflecting the sensitivity of cell status to intervention. The CVs calculated for the H9c2 group are 6.88, 0.26, 21.78, 7.54, 11.81, and 11.07, respectively; for the HeLa group, they are 13.88, 0.25, 28.12, 8.53, 17.89, and 14.09, respectively. It is evident that the CVs for the HeLa group are generally higher than those for the H9c2 group. The p-value from the paired T-test was 0.0242, indicating a statistically significant difference between the two groups (p < 0.05). Consequently, it can be concluded that, compared to cardiac myoblasts, cervical cancer cells demonstrate sensitivity to this concentration of cisplatin. This discrepancy may stem from the inherently faster proliferation rates of HeLa compared to H9c2, with cisplatin exhibiting increased cytotoxicity in rapidly dividing cellular contexts [87]. Furthermore, genetic and phenotypic distinctions between HeLa and H9c2, such as differences in cyclin-dependent kinase (CDKs) [88] activity and apoptotic protein (e.g., Bcl-2) [89] expression, may influence their respective susceptibilities to cisplatin.

## 4 Conclusion

In this work, we present a hyperstatic DL-driven AIV system, providing robust 3D single-cell imaging for high-resolution, label-free, and long-term cell fate observation. With the unique design of the lab on a four-pointed array chip, this AIV system enables stable rotation of single cells in-plane and out-of-plane, providing full-angle structural information for precise 3D reconstruction across uniform batches of controlled targets. Notably, we combined computer vision and DL techniques to analyze 3D image data, employing methods such as gray-level co-occurrence matrix, PCA, SVM, DDPM, RESNET50, etc., to elucidate inter-class differences among four cell types from different perspectives (with maximum group separation rate up to 98 %). The protocol affirms the accuracy of 3D reconstruction, offering an effective new paradigm for 3D data analysis and pattern recognition in future research. Moreover, the workflow of DL-driven video-input and 3D-output reconstruction significantly simplifies the tomographic imaging process, thereby increasing its accessibility for broader applications. By exposing various cell types into commonly used two drugs (POM and cisplatin), the easily obtainable target data were input into the established model, successfully reveal the long-term observation of cellular fate through spatiotemporal decoding.

In summary, this research innovatively integrates acoustic microfluidics with AI algorithms, with multi-degree-of-freedom rotation as the pivot and cell 3D reconstruction as the application, realizing precise spatiotemporal cell observation. Our research demonstrates an effective proof-of-concept platform for label-free rotational tomography of single cells, thereby expanding the horizons for the integration of AI algorithms within lab-on-a-chip platforms. This advancement holds substantial promise as a potent tool across numerous biological and medical domains, including but not limited to drug screening and precision oncology.

## CRediT authorship contribution statement

**Yuxin Wang:** Writing – original draft, Visualization, Validation, Software, Resources, Project administration, Methodology, Investigation, Formal analysis, Data curation, Conceptualization. **Shizheng Zhou:** Writing – original draft, Visualization, Validation, Software, Methodology, Investigation, Data curation, Conceptualization. **Yue Quan:** Resources, Methodology. **Yu Liu:** Visualization, Software, Methodology. **Bingpu Zhou:** Supervision. **Xiuping Chen:** Supervision. **Zhichao Ma:** Supervision, Resources. **Yinning Zhou:** Writing – review & editing, Visualization, Validation, Supervision, Resources, Project administration, Methodology, Investigation, Funding acquisition, Formal analysis, Data curation, Conceptualization.

## Declaration of Generative AI and AI-assisted technologies in the writing process

During the preparation of this work the author used ChatGPT 4 in order to improve readability and language. After using this tool, the author reviewed and edited the content as needed and takes full responsibility for the content of the publication.

## Declaration of competing interest

The authors declare that they have no known competing financial interests or personal relationships that could have appeared to influence the work reported in this paper.

## Data Availability

Data will be made available on request.
