# Supplementary material for: Label-free spatiotemporal decoding of single-cell fate via acoustic driven 3D tomography
Source: Mater Today Bio. 2024 Aug 13;28:101201. doi: 10.1016/j.mtbio.2024.101201 (PMC11364901; doi:10.1016/j.mtbio.2024.101201)
Supplement: Multimedia component 1 [file mmc1.docx]

**Supplementary material**

**Label-Free Spatiotemporal Decoding of Single-Cell Fate via Acoustic Driven 3D Tomography**

*Yuxin Wang^1^, Shizheng Zhou^1^, Yue Quan^1^, Yu Liu^1^, Bingpu Zhou^1^, Xiuping Chen^2^, Zhichao Ma^3^, Yinning Zhou^1*^*

1

Joint Key Laboratory of the Ministry of Education, Institute of Applied Physics and Materials Engineering, University of Macau, Avenida da Universidade, Taipa, Macau 999078, China

2

State Key Laboratory of Quality Research in Chinese Medicine, Institute of Chinese Medical Sciences, University of Macau, Avenida da Universidade, Taipa, Macau 999078, China

3

Institute of Medical Robotics, School of Biomedical Engineering, Shanghai Jiao Tong University, No.800 Dongchuan Road, Shanghai 200240, China

**Supplementary material abstract**

- Numerical simulations of microstreaming; Algorithm for measuring cell major axis; Calculation of the correlation coefficient between two images; Computation of grayscale distribution histograms; Feature engineering; Method for small-sample classification; Methods for data augmentation and large-sample classification; Microchip design and fabrication methods; Parameter optimization based on simulations; Rotations of B16 and MP using AIV system; 3D reconstruction of HeLa, B16, H9c2, and MP; Results of data augmentation and classification; Training progresses of B16 and MP; Live-dead staining of cells before and after rotation; Cross-validate the observed results with confocal microscopy
- Supplementary Video 1 Rotation of Cells
- Supplementary Video 2 3D View of a Single Cell

**1. Supplementary texts**

**1.1. Numerical simulations of microstreaming**

The finite element method (FEM) based simulations of low-frequency acoustic flow field were conducted using COMSOL Multiphysics 6.0 software. A 3D model was employed for these simulations.

Under external acoustic driving, liquid disturbances can be described using three variables: temperature $T$, pressure $p$, and velocity $v$. Considering first-order and second-order, the variables can be represented as: [1]

|  | ${T =T}_{0}+T_{1}+T_{2}$ | (1) |
| --- | --- | --- |

|  | $p = p_{0} +p_{1}+p_{2}$ | (2) |
| --- | --- | --- |

|  | $v = v_{1} +v_{2}$ | (3) |
| --- | --- | --- |

Where $T_{0}$and $p_{0}$represent the constant temperature and pressure before excitation, and $v$ on all walls.

The temperature for each boundary condition has not been considered. The first-order velocity $v_{1}$is used as a boundary condition to model the sound source. [2]

|  | $n\cdot v_{1}=v_{a}e^{-i\omega t}$ | (4) |
| --- | --- | --- |

Where $n$ is the normal vector, $v_{a}$is the magnitude of the normal velocity at the driving boundary, and $\omega$is the angular frequency characterizing the harmonic time dependency. Using the thermal-viscous acoustic module in the frequency domain, a first-order acoustic field can be obtained. The first-order equations can be represented as: [2]

|  | $i\omega T+\gamma D\nabla^{2}T=\frac{\gamma-1}{\alpha}\nabla\cdot v_{1}$ | (5) |
| --- | --- | --- |

|  | $i\omega\rho_{f}v_{1}+\mu\nabla^{2}v_{1}+\mu\left[ \beta+\frac{i}{\gamma k\mu\omega} \right]\nabla\left( \nabla\cdot v_{1} \right)=\frac{\alpha}{\gamma k}\nabla T$ | (6) |
| --- | --- | --- |

In the equation, *ω* represents angular frequency, *T* stands for temperature, *γ* denotes specific heat ratio, *D* is the thermal diffusion coefficient, $\rho_{f}$ is the fluid density, *μ* stands for fluid dynamic viscosity, *k* represents fluid compressibility, and *β* indicates viscosity ratio.

Based on the computed $v_{1}$ and $\rho_{1}$obtained from the first-order acoustic field, the laminar flow module is utilized to obtain the second-order acoustic field: [3]

|  | $\rho_{f}\nabla\cdot v_{2}=-\nabla\cdot\left( \rho_{1}v_{1} \right)$ | (7) |
| --- | --- | --- |

|  | $\mu\nabla^{2}\left\langle v_{2} \right\rangle+\beta\mu\nabla\left( \nabla\cdot\left\langle v_{2} \right\rangle\right)-\left\langle\nabla p_{2} \right\rangle=\left\langle\rho_{1}\partial_{t}v_{1} \right\rangle+\rho_{f}\left\langle\left( v_{1}\cdot\nabla\right)v_{1} \right\rangle$ | (8) |
| --- | --- | --- |

It can be observed that the second-order field on the left-hand side of the equation is determined by the first-order field on the right-hand side.

**1.2. Cell major axis measurement algorithm**

In order to characterize the rotational stability of cells, we employed an algorithm for calculating the major axis diameter of cells. [4] The algorithm involved several key steps. Initially, cell images were acquired using a microscope. These images underwent preprocessing, which included denoising, smoothing, and contrast enhancement to facilitate the subsequent identification of cell boundaries. The algorithm then employed edge detection techniques, Canny, to pinpoint the edges of the cells.

Following edge detection, the algorithm connected these edges into closed contours and extracted the contours outlining the cells. Subsequently, an ellipse fitting process, employing the least squares method, was used to fit the cell’s contour into an ellipse. Finally, the length of the major axis was measured by retrieving this information from the fitted ellipse. The results are as shown in the Figure 2d, f, S4.

**1.3. Calculating the correlation coefficient between two images**

In order to characterize the rotational stability of cells, we calculated the correlation coefficients between the first image and a subsequent series of images. Our aim was to observe periodic variations in the correlation coefficients, indicating the reproducibility of cell orientation over a complete rotation cycle. The process of calculating the correlation coefficient between two images involved several steps. [5] First, the images to be compared were obtained. Following that, necessary preprocessing steps were applied, including denoising and adjustments to brightness and contrast, ensuring accuracy in the subsequent comparison.

The images were then converted to grayscale to simplify calculations and ensure consistent grayscale information between them. The calculation of the correlation coefficient was carried out using the Pearson correlation coefficient. For two one-dimensional arrays, *x* and *y*, the Pearson correlation coefficient formula is as follows: [5]

|  | $r=\frac{\sum_{i=1}^{n} \left( x_{i}-\bar{x} \right)\left( y_{i}-\bar{y} \right)}{\sqrt{\sum_{i=1}^{n} \left( x_{i}-\bar{x} \right)^{2}}\sqrt{\sum_{i=1}^{n} \left( y_{i}-\bar{y} \right)^{2}}}$ | (9) |
| --- | --- | --- |

Here, $\bar{x}$ and $\bar{y}$ are the means of arrays *x* and *y*, and *n* is the length of the arrays.The interpretation of the result is based on the correlation coefficient’s range from $-1$ to 1, where $-1$ indicates perfect negative correlation, 1 indicates perfect positive correlation, and 0 indicates no correlation. The results are as shown in the Figure 2g-h and Figure S5.

**1.4. Computing the grayscale distribution histograms**

In order to characterize the morphological features of 3D reconstructed images of cells and demonstrate the separability of the four cell types, preparing for the subsequent classification, we separately computed and plotted the grayscale distribution histograms of cell nuclei and cell membranes for each of the four cell types. The algorithm for computing the grayscale distribution histograms involved several steps. Initially, the grayscale image was obtained from the image source. Then, it was converted to grayscale, achieved by averaging the channels for an RGB image or using weighted transformations.

The next step was to initialize a histogram array with 256 bins, each representing a grayscale level. Initially, all bins were set to zero. The algorithm then iterated through each pixel in the image, mapping its grayscale value to the nearest integer (typically in the range of 0 to 255), and incrementing the corresponding histogram bin.

Optionally, the histogram could be normalized by dividing each bin’s value by the total number of pixels in the image, providing the relative frequency of each grayscale level. [6]

|  | $P\left( i \right)=\frac{h_{i}}{N}$ | (10) |
| --- | --- | --- |

Here is the formula for computing the normalized histogram, where $h_{i}$represents the value of the *i* ^th^ bin, *N* is the total number of pixels in the image. The results are as shown in the Figure 3c and Figure S6-9.

**1.5. Feature engineering**

To characterize the cellular features of reconstructed 3D cell images, we employed image processing algorithms for feature extraction. In essence, we utilized a mean correlation threshold for binary segmentation of cell images, followed by segmentation of target regions. Subsequently, through the grayscale spatial correlation properties, we extracted the texture features of cells and calculated the entropy of the cell images. These features include contrast, dissimilarity, homogeneity, energy, correlation, and Shannon entropy. [4] Contrast, representing the degree of brightness variation in the image, reflects discernible changes in cellular nucleus and membrane surfaces; Dissimilarity, measuring the difference between adjacent pixels, can reveal heterogeneity in cell nucleus and membrane surfaces in the cell images; Homogeneity, indicating the uniformity of pixel intensity distribution in the image, reflects the uniformity of cell structures in the cell images; Energy, reflecting the statistical energy of texture in the image, describes the overall strength of cell texture in the images; Correlation, measuring the linear relationship between pixels in the image, can be employed to analyze the correlation between structures such as cellular nucleus and membrane surfaces; Shannon entropy, used to measure the uncertainty of information in the image, reflects the complexity of cell images.

Through these characteristics, we comprehensively characterized the uniformity and complexity of cellular nucleus, membrane surfaces, and local textures. The processes of target region segmentation and morphological feature value extraction were carried out using the OpenCV and Scikit-image libraries. The results are as shown in the Figure 3g.

**1.6. Small-sample classification**

In order to demonstrate the distinctiveness among the four cell types, we further employed the Support Vector Machine (SVM) algorithm. [7] The dataset comprises four cell types, each consisting of grayscale distribution histogram data from ten images of 3D reconstruction. For each cell type, five images correspond to cell nuclei, and the remaining five images correspond to cell membranes. The algorithm for four-class classification involved several key steps. Initially, a labeled dataset containing instances of the four classes was collected and prepared. Relevant features were extracted from each sample to capture crucial aspects of the data.

The dataset was then partitioned into training and testing sets to facilitate the independent evaluation of the model’s performance. The SVM classifier was trained using the training set.

Upon evaluating the model’s performance, results were interpreted to understand its classification accuracy for each class and its generalization capabilities. The SVM proved to be a robust classifier and the higher accuracy provides evidence that SVM is suitable for small-sized dataset. The results are as shown in the Figure S12.

**1.7. Data augmentation and large-sample classification**

The subtle differences in features among the four cell populations prompted us to enhance cell recognition by constructing a deep learning model with effective feature extraction. Among numerous deep learning models, ResNet has been widely employed for feature extraction. Leveraging residual connections, ResNet can preserve the original features of images. [8]

To ensure an ample number of learning samples, we applied data augmentation methods to the images, including brightness enhancement, image flipping, and rotation. Furthermore, to prevent overfitting of the model to the originally collected cell images during training, we innovatively employed image generation methods. We trained the DDPM (Denoising Diffusion Probabilistic Model) [9] to augment images, generating large numbers of new images within the feature range for each class subset. The resolution of the generated images was relatively lower than that of the original images. In total, we obtained 1000 images, with an equal number in each subset of the training set. Presented in the Figure S10.

The dataset was shuffled and split into training and testing sets in an 8:2 ratio. We utilized the PyTorch framework to build the ResNet50 model, and training and blind testing were conducted on a PC with an i9-11900K CPU, 64 GB of RAM, and an Nvidia GeForce GTX 3090 GPU. During the training phase, the model employed the adaptive moment estimation (Adam) [10] optimizer with a learning rate of 0.01 to update the network’s weights and biases over 200 epochs. Following the application of 5-fold cross-validation, a Stacking Ensemble methodology was employed to generate the ultimate model. Specifically, throughout the training phase, a meticulous 5-round cross-validation process was executed, with the paramount weights, indicative of superior performance in each iteration, meticulously preserved. These optimal weights were subsequently employed as the foundational models for the Stacking Ensemble. Subsequently, leveraging the predictions from these foundational models as inputs, an ensemble meta-model was trained, contributing to a further augmentation of overall performance. Through the adept utilization of Stacking Ensemble, the amalgamation of strengths from each foundational model facilitated the creation of a more robust and formidable ultimate model. The results are shown in the Figure S10-12.

**1.8. Cross-validate the reconstruction results with confocal microscopy**

We calculated histograms and cumulative distribution functions (CDFs) of grayscale values for nuclei and cell membranes of four types of cells predicted by 3D-CNN, and compared these with confocal images. As shown in Figures S16 and S17, the grayscale histograms and CDF curves of 3D-CNN closely align with the confocal ground truth, indicating that our method’s 3D reconstructions are highly consistent with the labeled data generated by confocal microscopy. This highlights the robustness of our model. To further validate the consistency between the regression predicted data and confocal data, we used the morphological parameters of H9c2 cells treated with cisplatin (contrast, correlation, energy, homogeneity, dissimilarity, and entropy) changing over time as examples. We calculated these six parameters from both sets of image data and performed statistical analysis using paired t-tests: p1 = 0.0521, p2 = 0.3068, p3 = 0.3978, p4 = 0.1588, p5 = 0.1759, p6 = 0.0562 (all p-values greater than 0.05), indicating no significant statistical difference between the two data sets, thus proving the robustness of our model. (Figure S18)

**References**

[1] P.B. Muller, R. Barnkob, M.J.H. Jensen, H. Bruus, A numerical study of microparticle acoustophoresis driven by acoustic radiation forces and streaming-induced drag forces, Lab on a Chip 12(22) (2012) 4617-4627.

[2] Y. Liu, Z. Wen, Z. Huang, Y. Wang, Z. Chen, S. Lai, S. Chen, Y. Zhou, Liquid Phase Graphene Exfoliation with a Vibration-Based Acoustofluidic Effector, Micromachines 14(9) (2023) 1718.

[3] Z. Ma, Y. Zhou, F. Cai, L. Meng, H. Zheng, Y. Ai, Ultrasonic microstreaming for complex-trajectory transport and rotation of single particles and cells, Lab on a Chip 20(16) (2020) 2947-2953.

[4] K.R. Castleman, Digital image processing, Prentice Hall Press1996.

[5] R. Szeliski, Computer vision: algorithms and applications, Springer Nature2022.

[6] T. Acharya, A.K. Ray, Image processing: principles and applications, John Wiley & Sons2005.

[7] W.S. Noble, What is a support vector machine?, Nature biotechnology 24(12) (2006) 1565-1567.

[8] K. He, X. Zhang, S. Ren, J. Sun, Deep residual learning for image recognition, Proceedings of the IEEE conference on computer vision and pattern recognition, 2016, pp. 770-778.

[9] J. Ho, A. Jain, P. Abbeel, Denoising diffusion probabilistic models, Advances in neural information processing systems 33 (2020) 6840-6851.

[10] D.P. Kingma, J. Ba, Adam: A method for stochastic optimization, arXiv preprint arXiv:1412.6980 (2014).

**2.** **Supplementary figures**


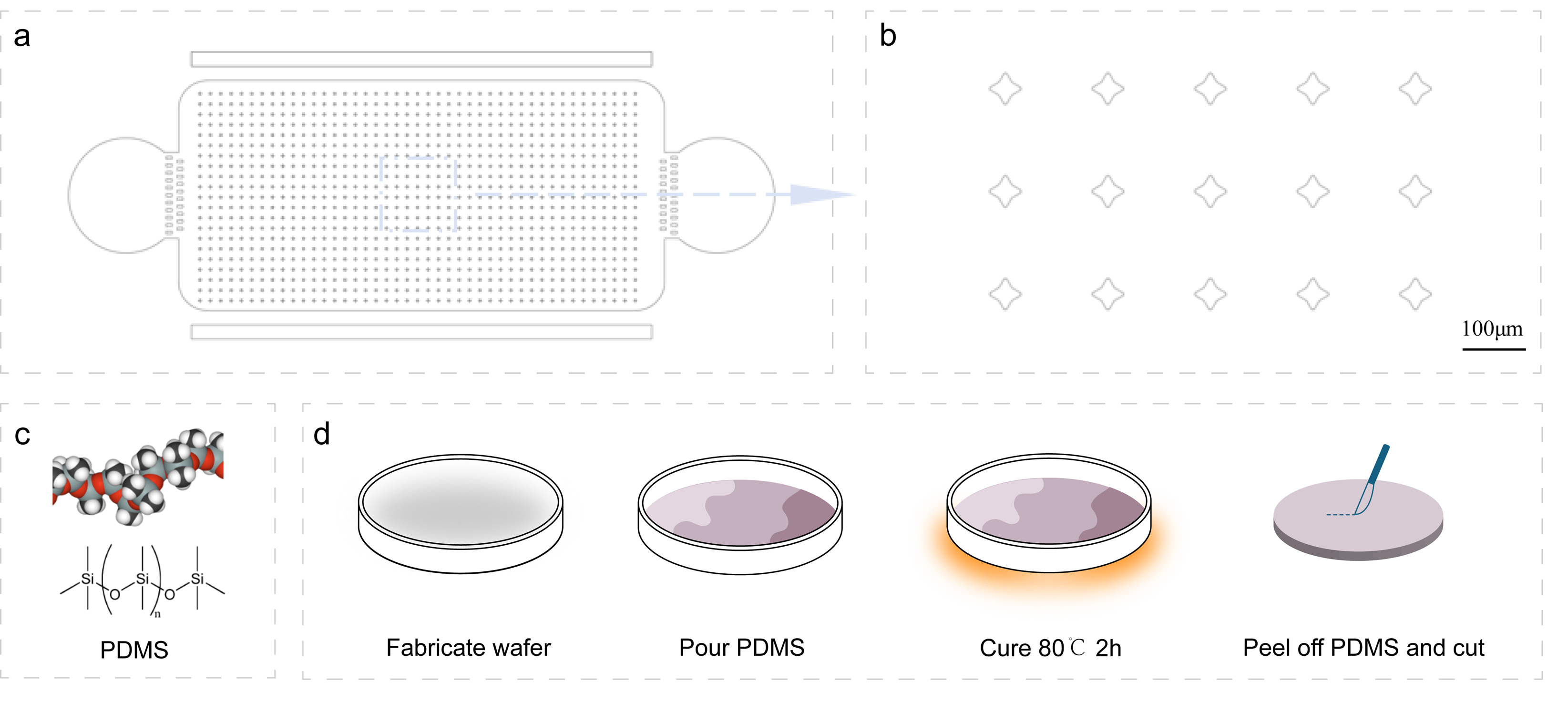


Figure S1. Microchip patterns with arrayed four-pointed stars drawn using CAD and fabricated with PDMS. a) Overview of the entire chip. b) Local magnification. Scale bar: 100µm. c) Structural formula of PDMS. d) Fabrication process of micropillars.


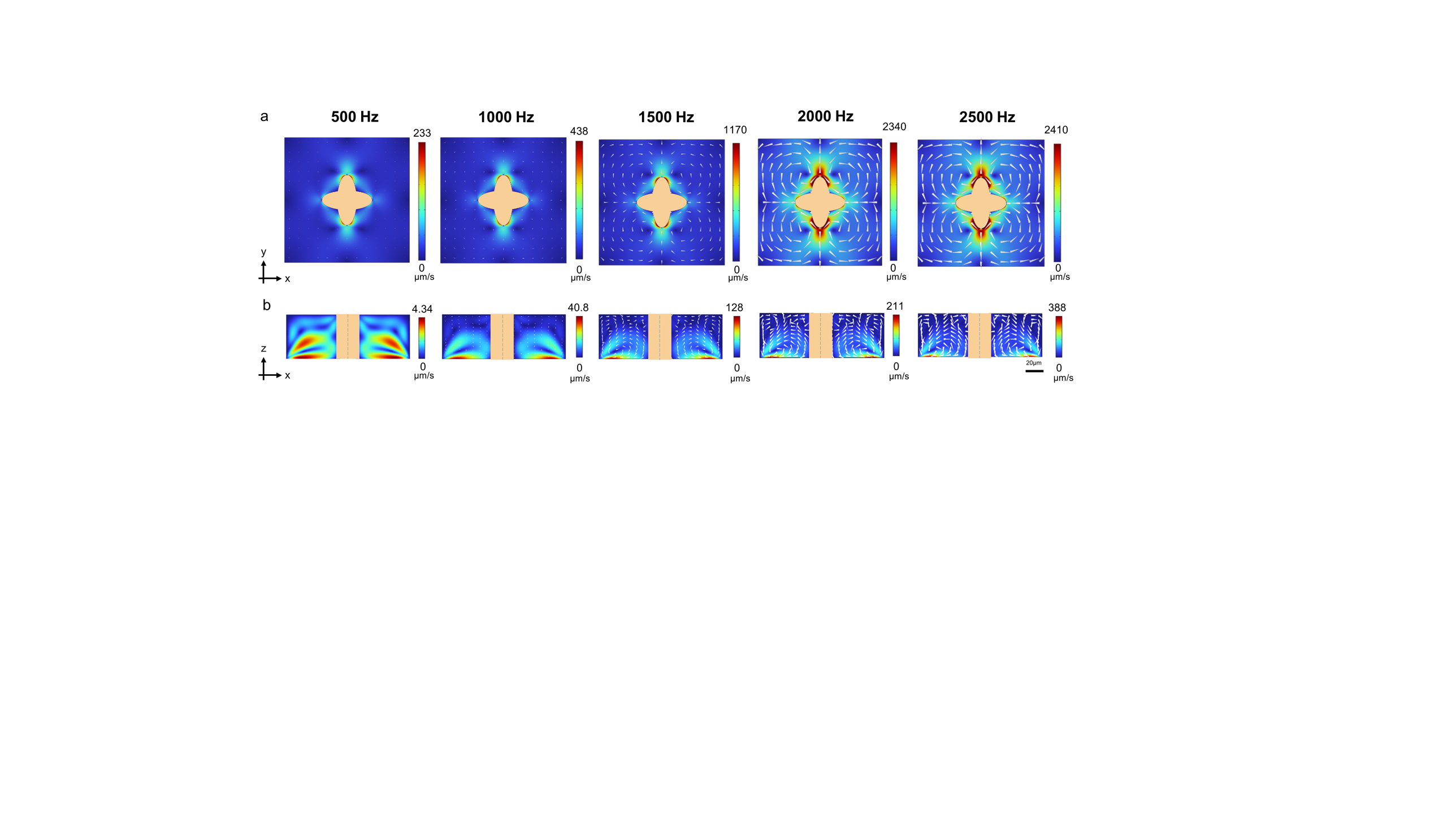


Figure S2. Optimization of vibration frequency parameters in simulations. a) Simulations of the velocity field under X-axis vibration across frequencies of 500 Hz, 1000 Hz, 1500 Hz, 2000 Hz, 2500 Hz, observed on the X-Y plane. Scale bar: 20µm. b) Simulations of the velocity field under Z-axis vibrations across above frequencies, observed on the X-Z plane. Scale bar: 20µm.


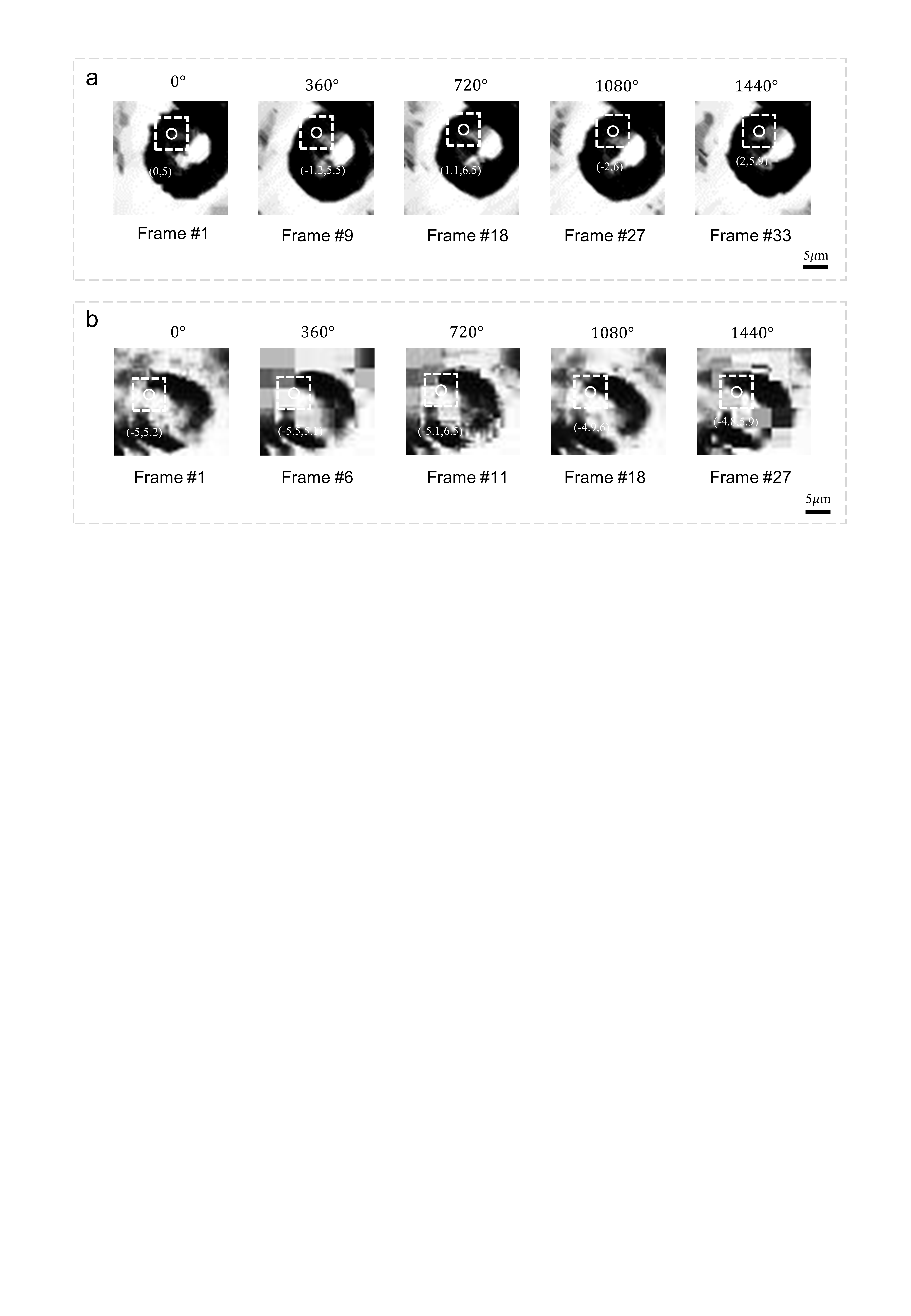


Figure S3. a) Out-of-plane rotation of B16 cells induced by Z-axis vibration is recorded, with feature points tracked in videos. Images are captured at each 360° rotation increment. Scale bar: 5µm. b) Out-of-plane rotation of MP cells induced by Z-axis vibration is recorded, with feature points tracked in videos. Images are captured at each 360° rotation increment. Scale bar: 5µm.


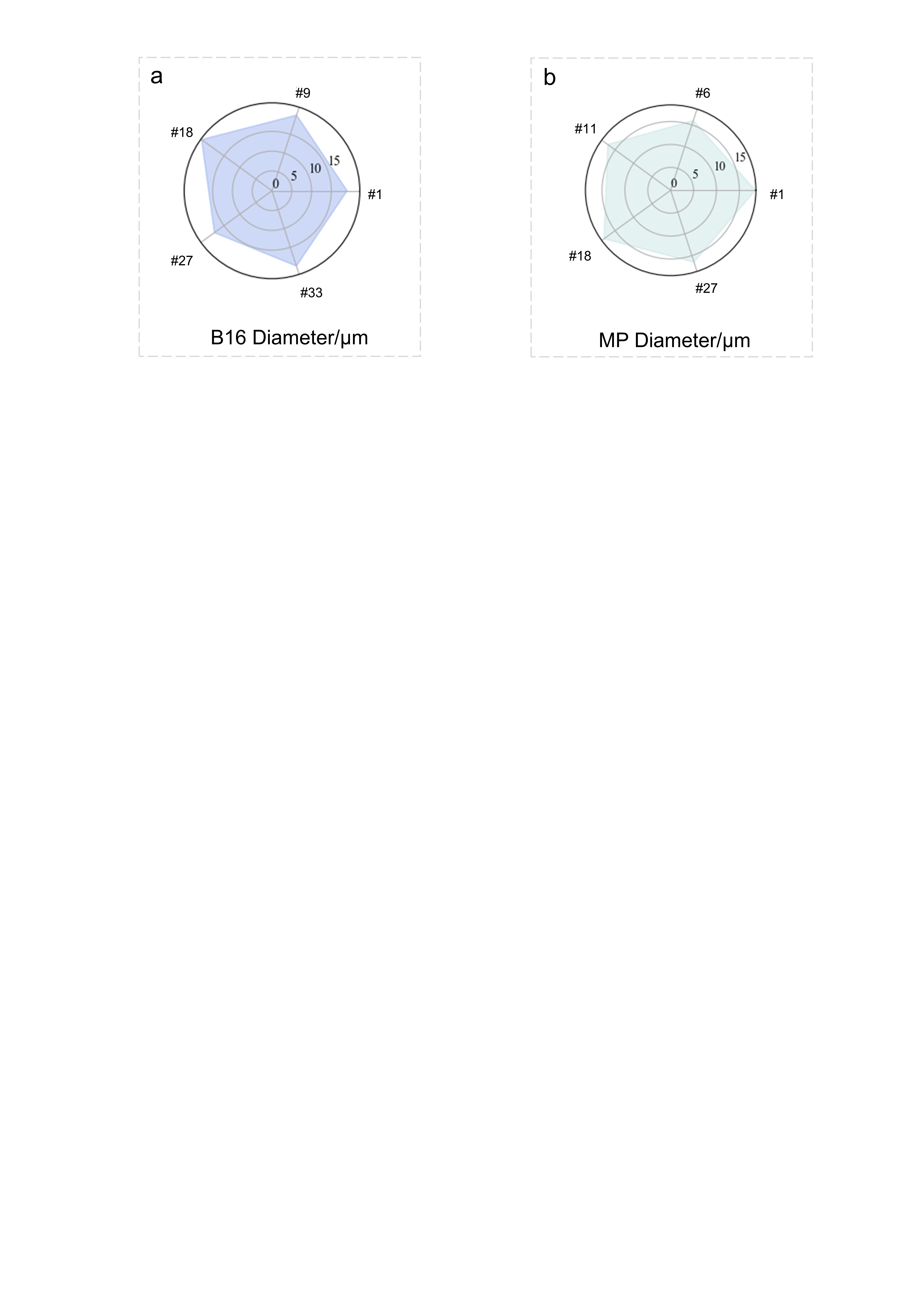


Figure S4. a) Calculation of the major axis diameter of B16 cells in five frames from Figure S3 a. A radar chart is plotted with axis labels representing frame numbers and data points representing the major axis diameter in µm. b) Calculation of the major axis diameter of MP cells in five frames from Figure S3 b. A radar chart is plotted with axis labels representing frame numbers and data points representing the major axis diameter in µm.


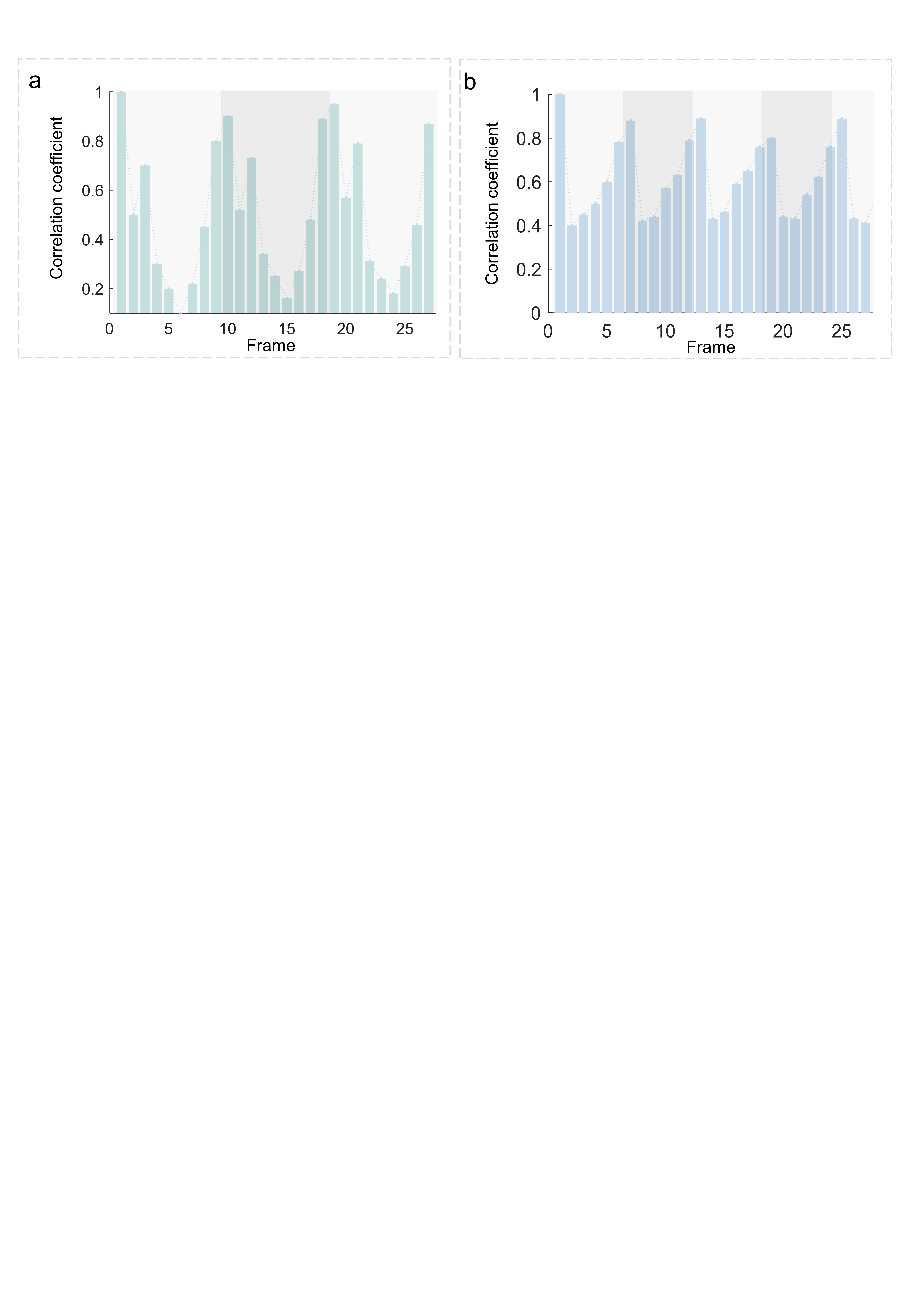


Figure S5. a) Correlation analysis of consecutive frames in the recorded rotation videos of B16. b) Correlation analysis of consecutive frames in the recorded rotation videos of MP. Histograms are plotted, with the horizontal axis representing frame numbers and the vertical axis representing correlation coefficients.


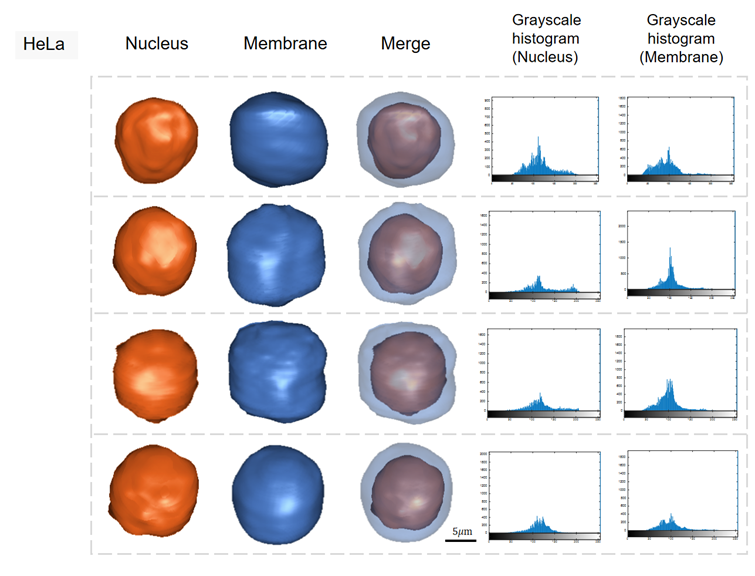


Figure S6. 3D reconstruction results of the cell nuclei, membranes and merged for HeLa of four experiments. Scale bar: 5µm. Grey-scale distribution histograms are plotted for the 3D reconstructed images of cell nuclei and membranes.


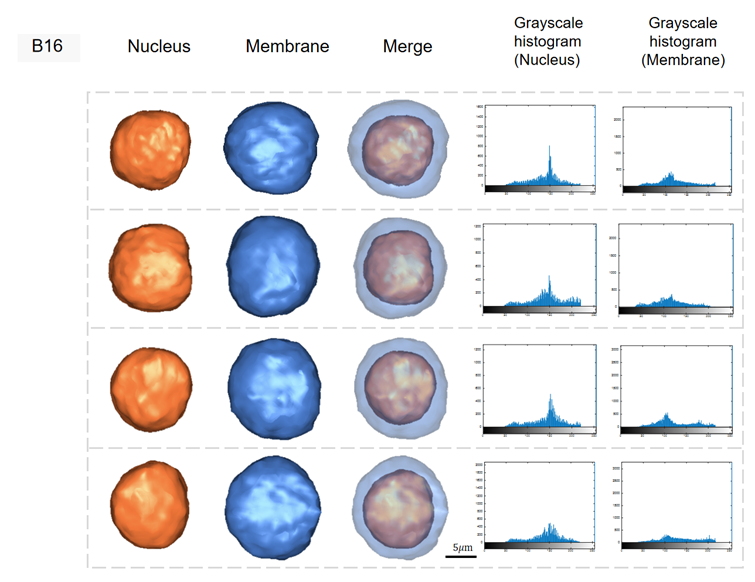


Figure S7. 3D reconstruction results of the cell nuclei, membranes and merged for B16 of four experiments. Scale bar: 5µm. Grey-scale distribution histograms are plotted for the 3D reconstructed images of cell nuclei and membranes.


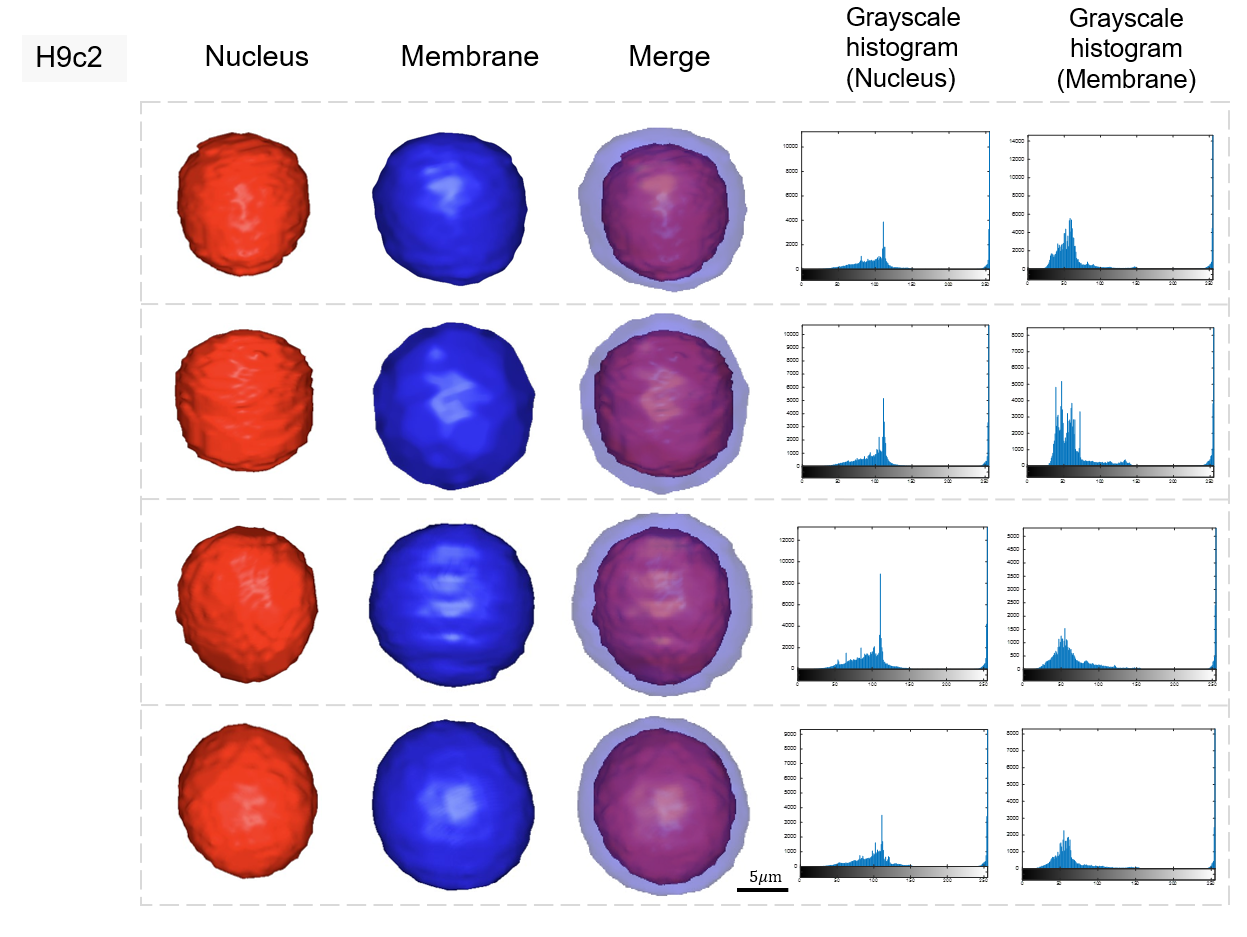


Figure S8. 3D reconstruction results of the cell nuclei, membranes and merged for H9c2 of four experiments. Scale bar: 5µm. Grey-scale distribution histograms are plotted for the 3D reconstructed images of cell nuclei and membranes.


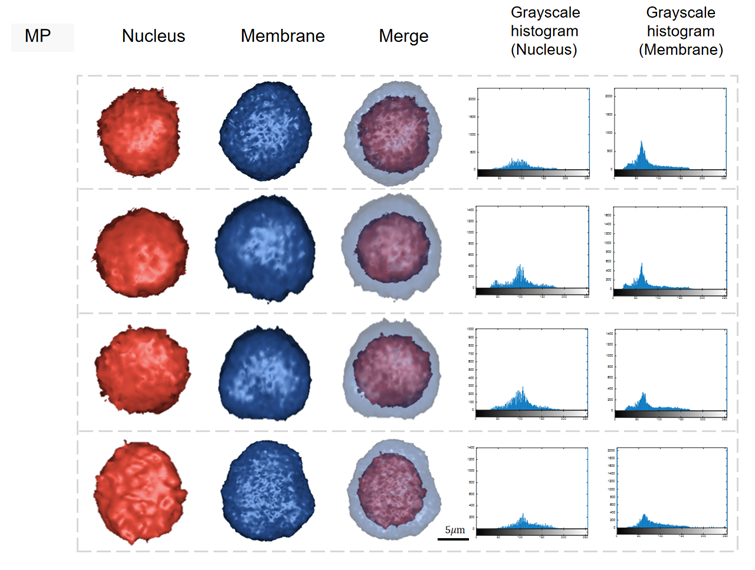


Figure S9. 3D reconstruction results of the cell nuclei, membranes and merged for MP of four experiments. Scale bar: 5µm. Grey-scale distribution histograms are plotted for the 3D reconstructed images of cell nuclei and membranes.


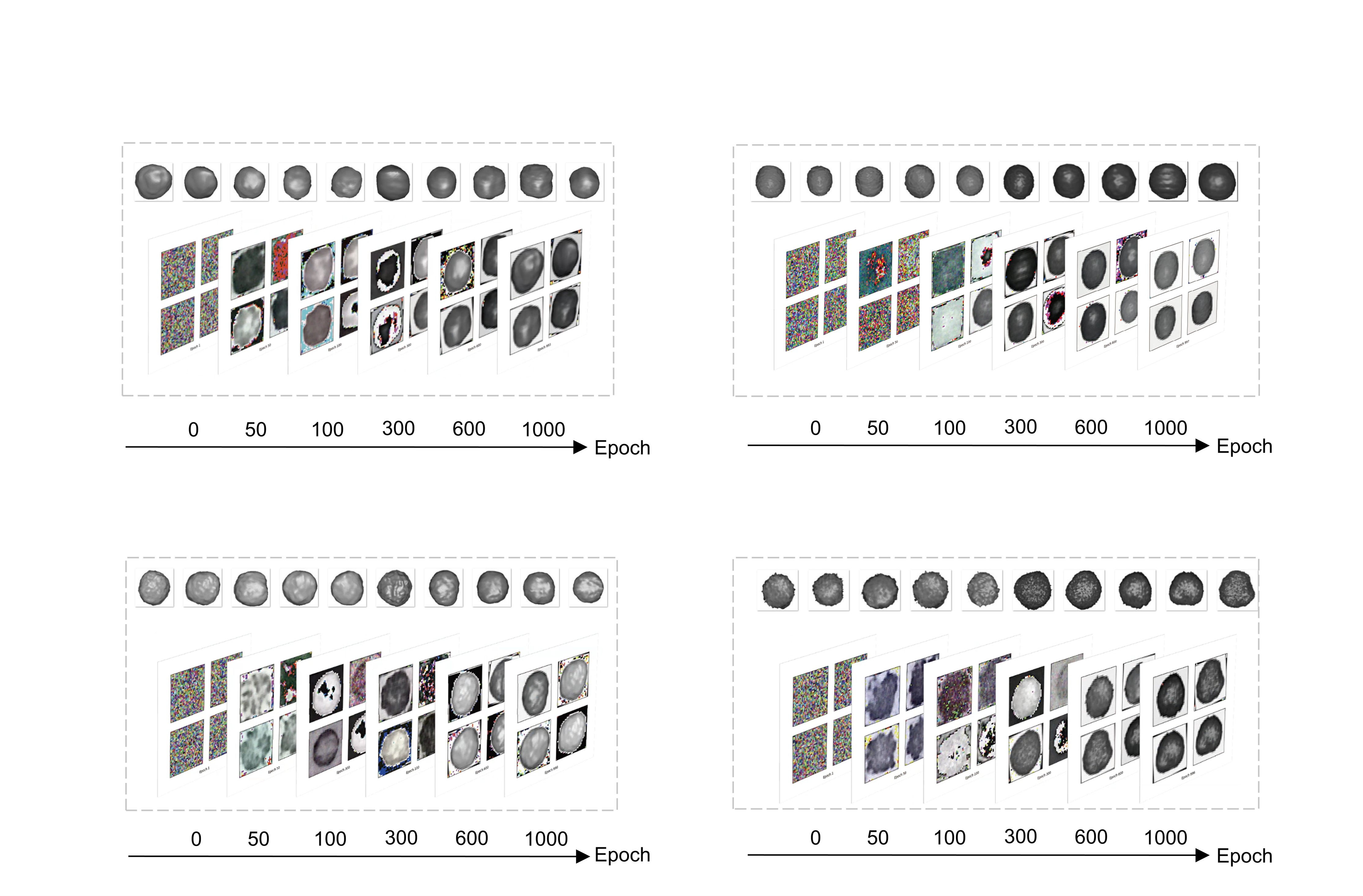


Figure S10. The process of images augment for four-type cells generated through Denoising Diffusion Probabilistic Model (DDPM).


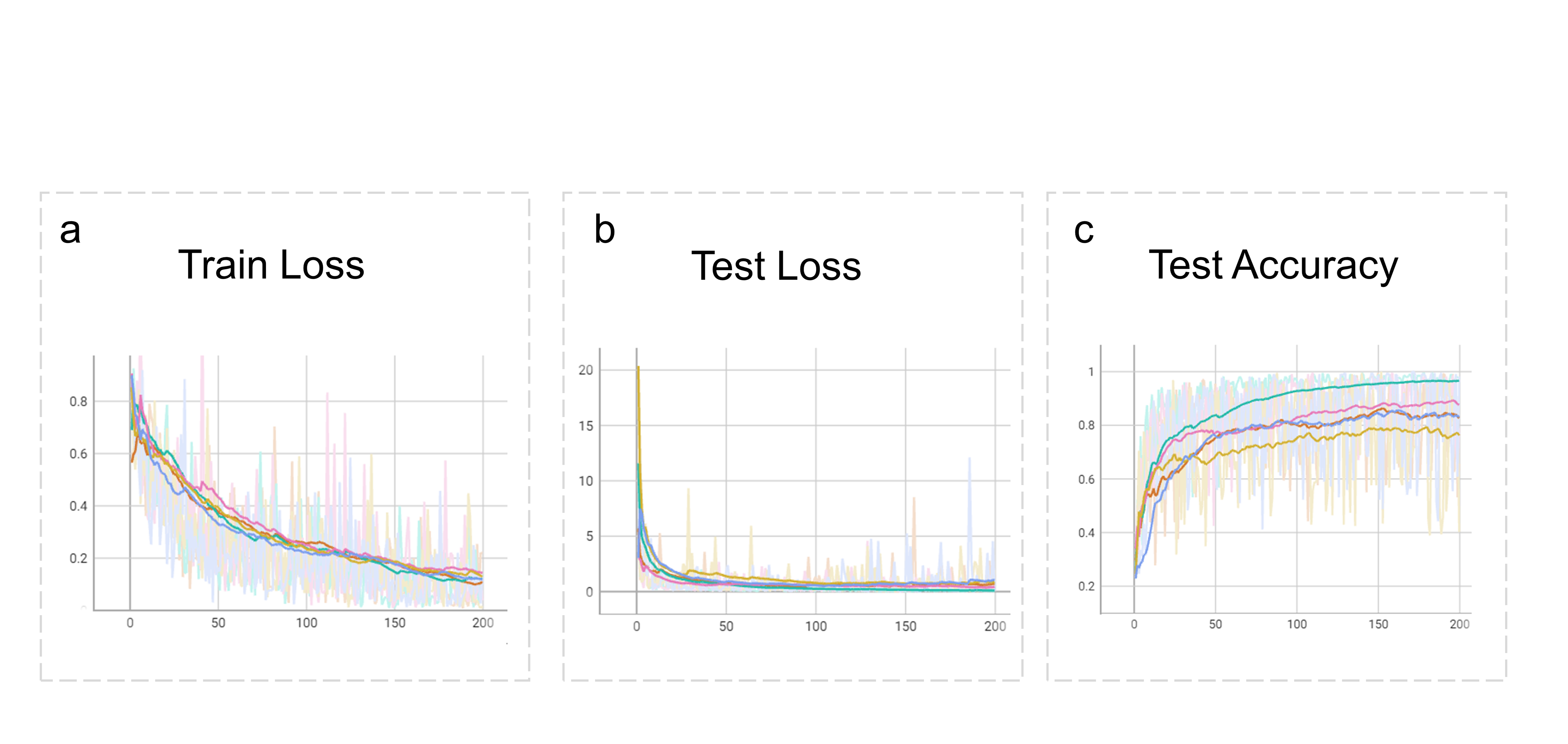


Figure S11. Classification of four cell types based on a big dataset. a) Training loss using RESNET50 classification method. b) Test loss using RESNET50 classification method. c) Test accuracy using RESNET50 classification method.


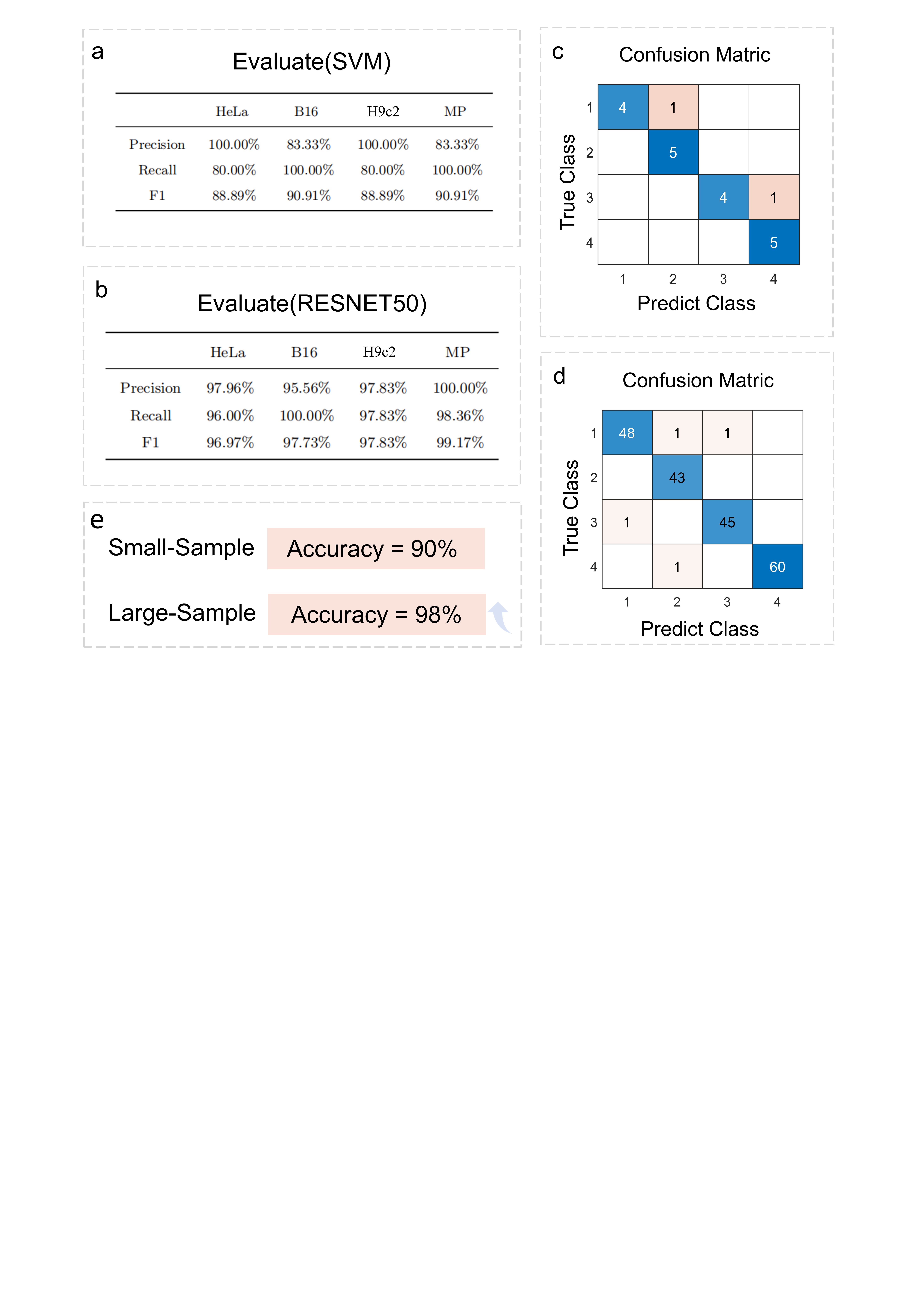


Figure S12. Classification of four cell types based on a small-sample and large-sample dataset. a) Algorithm evaluation for SVM four-class classification: precision, recall, F1 score, and accuracy. b) Algorithm evaluation for RESNET50 four-class classification: precision, recall, F1 score, and accuracy. c) Confusion matrix for SVM four-class classification. d) Confusion matrix for RESNET50 four-class classification. e) The classification accuracy is improved for large-sample datasets compared to small-sample datasets.


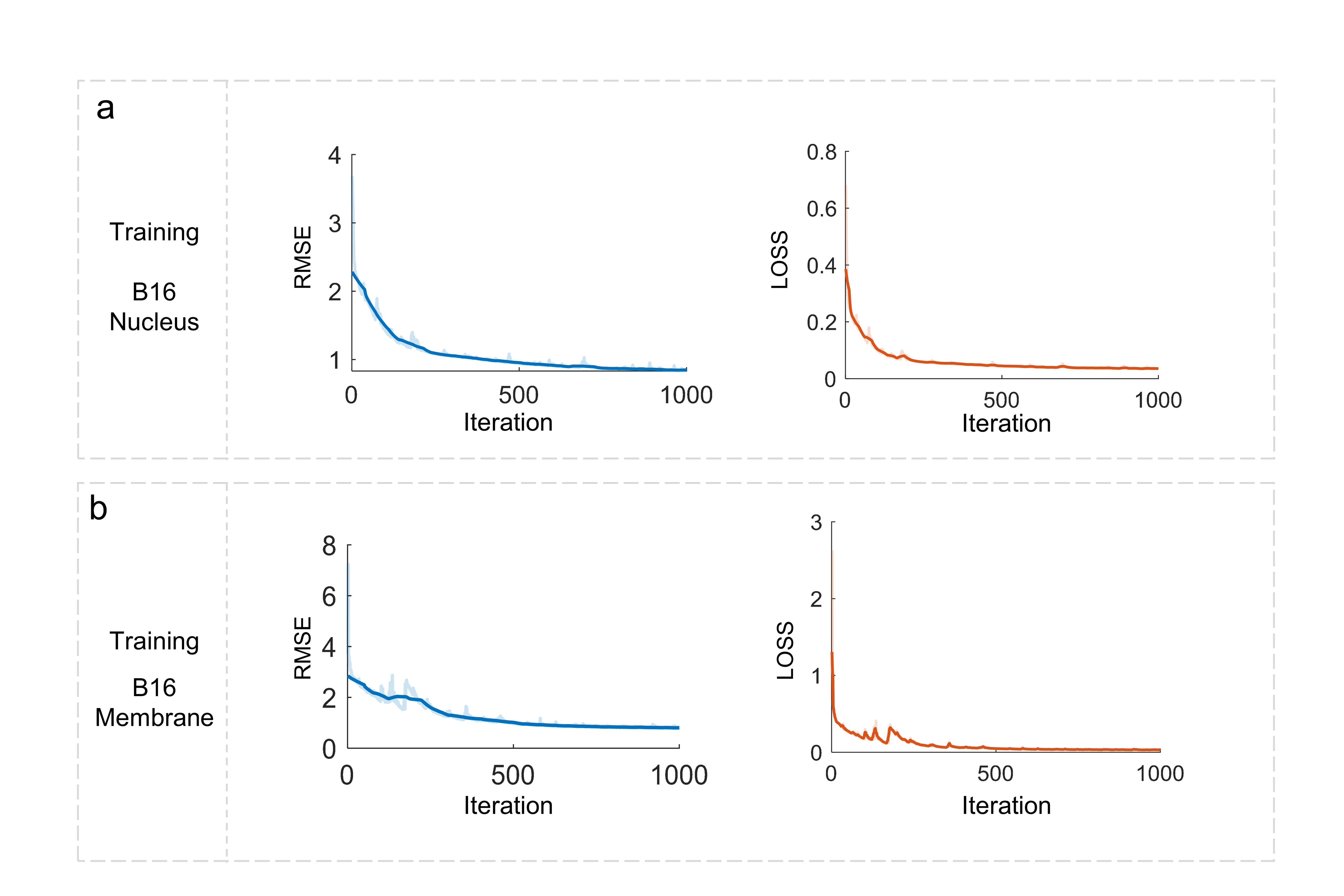


Figure S13. a) Training progress of root mean squared error (RMSE) and loss for nuclei of B16 cells using 3D convolutional neural network (3D-CNN) regression. b) Training progress of RMSE and loss for membranes of B16 cells using 3D-CNN regression.


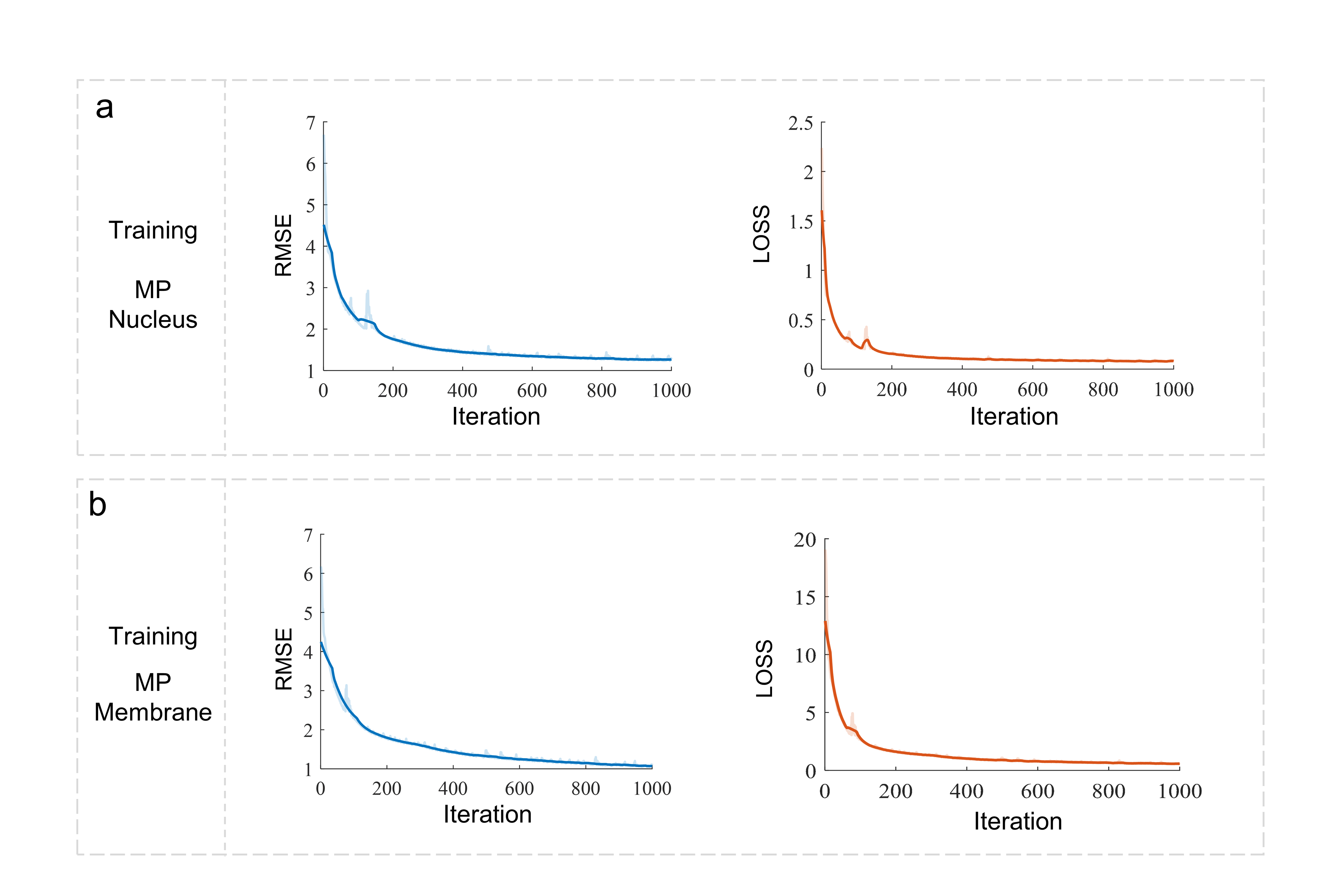


Figure S14. a) Training progress of RMSE and loss for nuclei of MP cells using 3D-CNN regression. b) Training progress of RMSE and loss for membranes of MP cells using 3D-CNN regression.


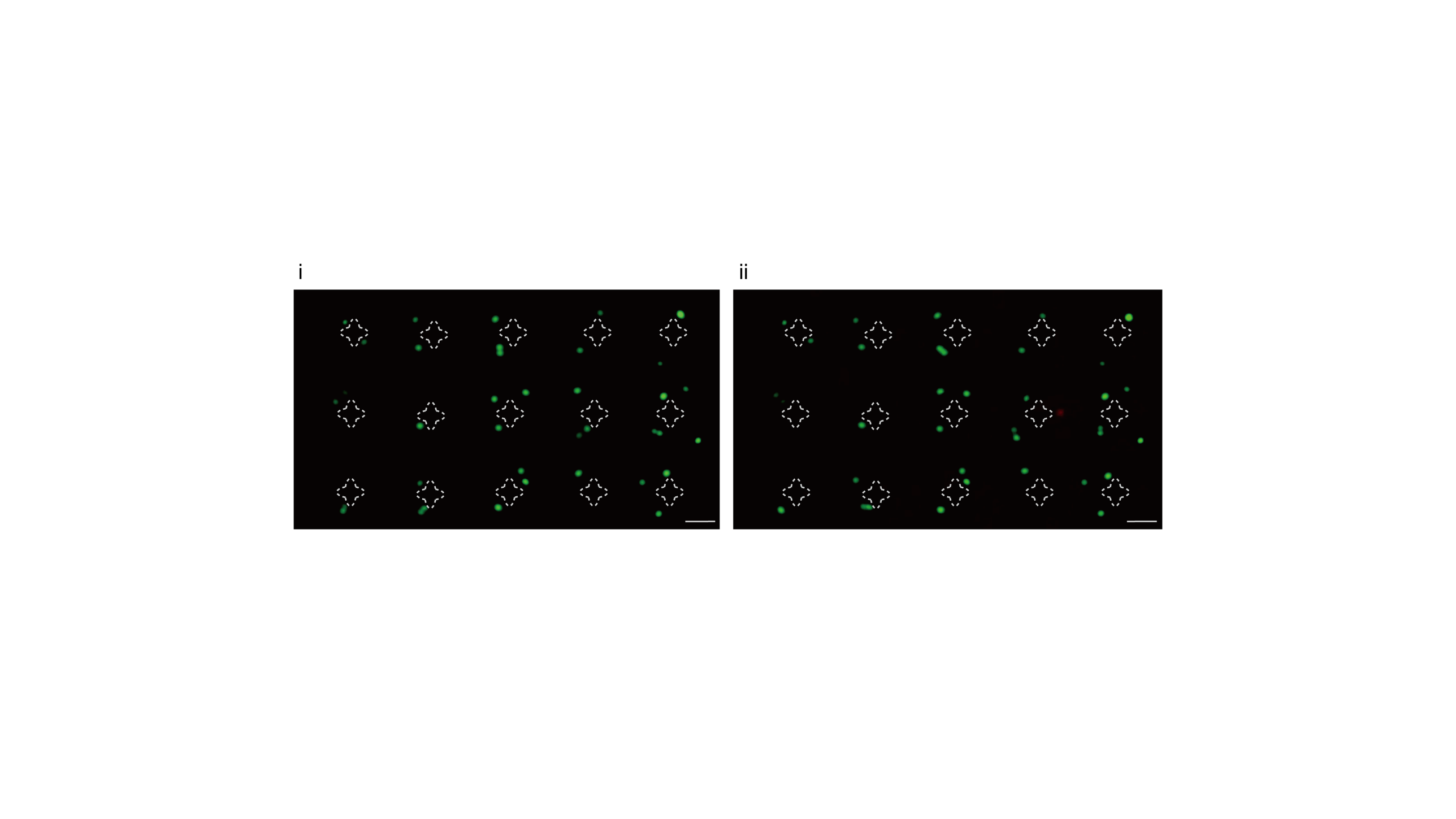
Figure 15. Live-dead staining of HeLa cells before and after rotation. Green fluorescence indicates live cells, while red fluorescence represents dead cells. i) Before rotation; ii) After rotation. Scale bar: 100 μm.





Figure S16. The 3D-CNN predicts the grayscale histograms and cumulative distribution functions (CDFs) of the nuclei for four types of cells, which are compared with confocal images.





Figure S17. The 3D-CNN predicts the grayscale histograms and cumulative distribution functions (CDFs) of the cell membranes for four types of cells, which are compared with confocal images.


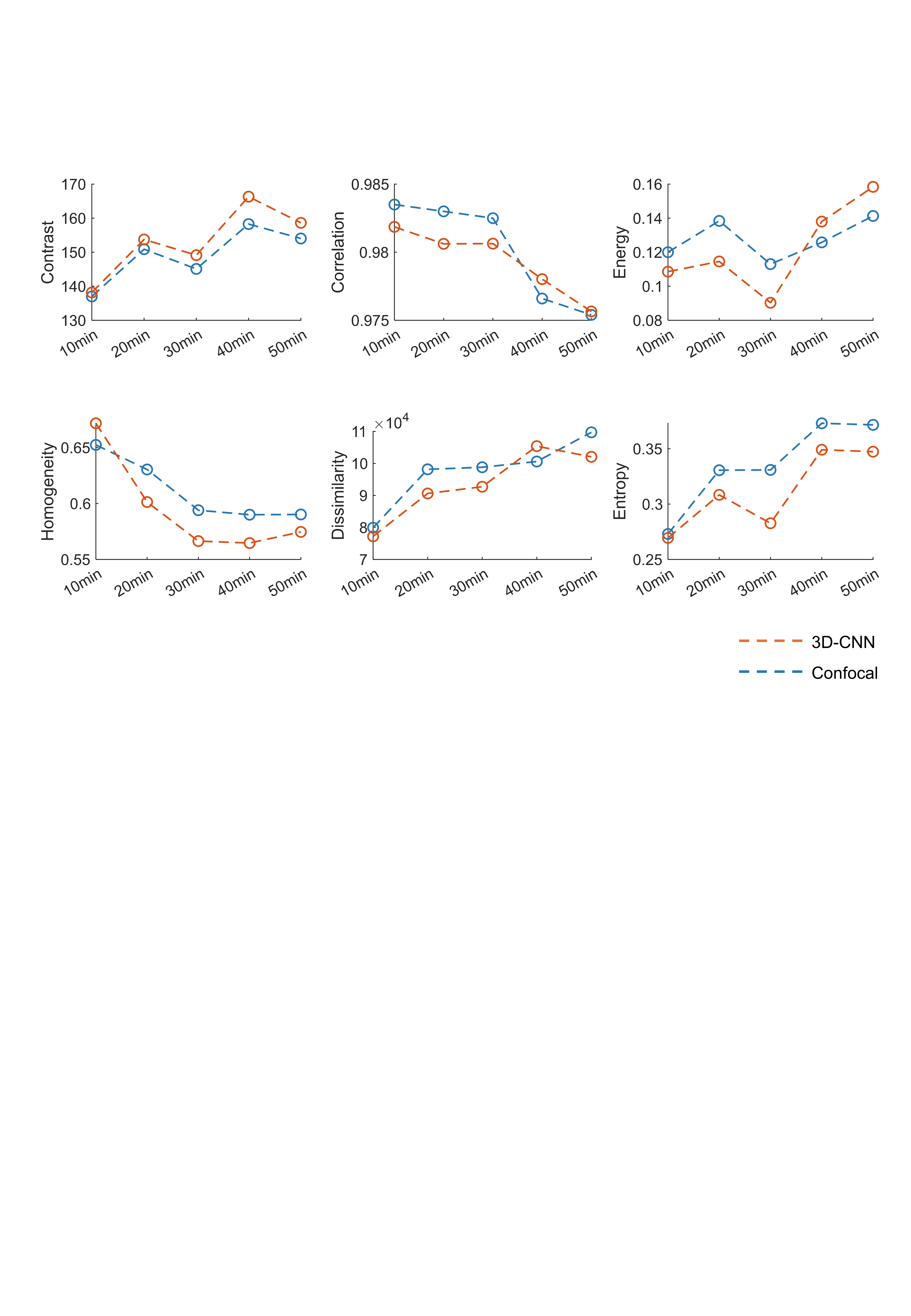


Figure S18. Graph of the changes in morphological parameters over time in H9c2 cells after treatment with cisplatin, including 3D-CNN and confocal microscopy.
